# Coordinated immune dysregulation in juvenile dermatomyositis revealed by single-cell genomics

**DOI:** 10.1172/jci.insight.176963

**Published:** 2024-05-14

**Authors:** Gabrielle Rabadam, Camilla Wibrand, Emily Flynn, George C. Hartoularos, Yang Sun, Chioma Madubata, Gabriela K. Fragiadakis, Chun Jimmie Ye, Susan Kim, Zev J. Gartner, Marina Sirota, Jessica Neely

**Affiliations:** 1 UC Berkeley-UC San Francisco Graduate Program in Bioengineering, and; 2 Department of Pharmaceutical Chemistry, UCSF, San Francisco, California, USA.; 3 Aarhus University, Aarhus, Denmark.; 4 Division of Pediatric Rheumatology, Department of Pediatrics,; 5 CoLabs,; 6 Graduate Program in Biological and Medical Informatics,; 7 Division of Rheumatology, Department of Medicine,; 8 Institute for Human Genetics,; 9 Department of Epidemiology and Biostatistics, and; 10 Bakar Computational Health Sciences Institute, UCSF, San Francisco, California, USA.; 11 Chan Zuckerberg Biohub, San Francisco, California, USA.; 12 Parker Institute for Cancer Immunotherapy, San Francisco, California, USA.; 13 Department of Pediatrics, UCSF, San Francisco, California, USA.

**Keywords:** Autoimmunity, Autoimmune diseases, Bioinformatics, Rheumatology

## Abstract

Juvenile dermatomyositis (JDM) is one of several childhood-onset autoimmune disorders characterized by a type I IFN response and autoantibodies. Treatment options are limited due to an incomplete understanding of how the disease emerges from dysregulated cell states across the immune system. We therefore investigated the blood of patients with JDM at different stages of disease activity using single-cell transcriptomics paired with surface protein expression. By immunophenotyping peripheral blood mononuclear cells, we observed skewing of the B cell compartment toward an immature naive state as a hallmark of JDM at diagnosis. Furthermore, we find that these changes in B cells are paralleled by T cell signatures suggestive of Th2-mediated inflammation that persist despite disease quiescence. We applied network analysis to reveal that hyperactivation of the type I IFN response in all immune populations is coordinated with previously masked cell states including dysfunctional protein processing in CD4^+^ T cells and regulation of cell death programming in NK cells, CD8^+^ T cells, and γδ T cells. Together, these findings unveil the coordinated immune dysregulation underpinning JDM and provide insight into strategies for restoring balance in immune function.

## Abstract 2

Disease heterogeneity in juvenile dermatomyositis is underpinned by coordinated dysregulation across the immune system at the cellular level.

## Graphical abstract

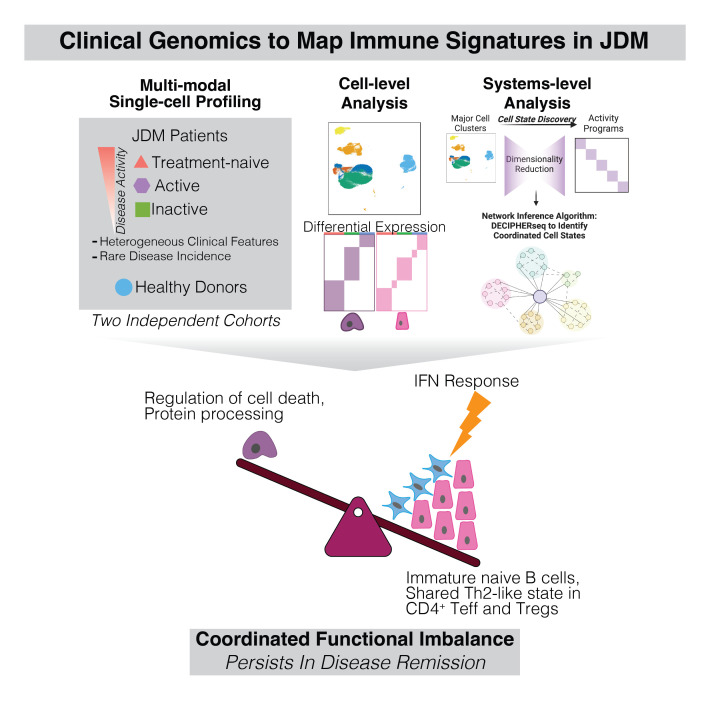

## Introduction

Juvenile dermatomyositis (JDM) is part of a broad group of childhood-onset autoimmune conditions characterized by a type I IFN gene signature and specific autoantibodies ranging from related systemic conditions such as systemic lupus erythematosus (SLE) to endocrine-specific disorders such as type 1 diabetes (1–3). Despite a shared IFN signature, JDM is associated with pathognomonic rashes and proximal muscle weakness resulting in distinct clinical phenotypes. The etiology of JDM is not fully understood, but studies have shown that JDM is autoimmune mediated and associated with a combination of genetic and environmental risk factors (4). While mortality is low with corticosteroid treatment, long-term patient follow-up studies have reported that 60%–70% of patients have cumulative tissue damage, with the risk of damage increasing almost linearly for each year after diagnosis (5–7). This finding highlights the importance of early disease intervention and the need for a personalized approach to disease management to improve upon these outcomes.

Clinical management of JDM currently relies on compiled empirical metrics such as physician global visual analog scale (VAS) of disease activity and muscle strength quantified via the childhood myositis assessment scale (CMAS) or manual muscle testing (MMT) (8). However, how these clinically observable phenotypes are rooted in disease immunopathology remains insufficiently understood. The presence of myositis-specific antibodies (MSA) that correspond to distinct clinical phenotypes and recent work showing that MSAs may be pathogenic suggest the involvement of B cells (9–11). The expansion of naive B cells in JDM has been highlighted by 3 independent studies using flow cytometry, mass cytometry, and single-cell RNA-Seq (12–14). The adaptive arm of the immune system is further implicated in disease pathogenesis by several large immunophenotyping studies that demonstrated the expansion of extrafollicular Th2 memory cells and central memory B cells (15, 16). Additionally, the innate immune system has emerged as a contributor in JDM with increased macrophages in skin and NK cell dysfunction described peripherally (13, 17, 18). Together, these findings highlight the involvement of both the adaptive and innate immune compartments in JDM in blood and disease-affected tissues. However, it also raises the question of whether the cause of JDM lies in a single cell type or is a manifestation of broadly dysregulated cellular interactions across the immune system.

Systems-level studies based on single-cell measurements are required to reveal how dysregulated cell populations act individually or cooperatively to produce the observed inflammation. Accordingly, several groups have turned to next-generation sequencing, as it enables unbiased profiling of tissues at a single-cell resolution. We previously described a pan–cell type IFN gene signature overexpressed in treatment-naive JDM that was most strongly correlated with disease activity in cytotoxic cell types (14). This signature has since been independently identified in the peripheral blood of treatment-naive patients (19). However, these studies have utilized small cohorts and lack pediatric controls. Thus, it has been challenging to determine which of these findings are specific to JDM compared with healthy children, how these disease-specific dysregulated cell states are coordinated with one another, and which of these states cooperatively change in response to treatment.

In this study, we addressed this challenge by profiling JDM across several stages of disease activity using multiplexed Cellular Indexing of Transcriptomes and Epitopes by sequencing (CITEseq) of peripheral blood mononuclear cells (PBMCs) from 15 patients with JDM, totaling 22 samples, and 5 healthy controls (HC). Compositional analysis of immune populations identified a disease activity–associated imbalance of naive and mature lymphocytes, corroborated by distinct immunophenotypes in treatment-naive disease. To move beyond the identification of disease-associated cell populations and toward an understanding of immune-scale dysregulation in JDM, we applied a recently developed computational method DECIPHERseq to infer networks of coordinated cell states from large cohorts of single-cell data (20). Importantly, this unsupervised method takes advantage of the biological heterogeneity in the entire data set, improving upon previous work that relied on pairwise comparisons of subsetted disease groups. Among other signatures previously masked by traditional single-cell analysis, this approach revealed cooccurring cell states in CD4^+^ T and B cell populations, suggestive of extrafollicular responses. A subset of these CD4^+^ T signatures implicates disruption of protein targeting and immune tolerance processes; notably, these cell states persist even in patients in remission off medication. Furthermore, we show that the hyperactive type I IFN response in disease is paralleled by impaired cell death processes in cytotoxic immune cells, highlighting the functional imbalance across immune compartments that typifies this complex autoimmune disease. This broadened understanding of the underlying immune dysregulation in disease can inform precision treatment strategies for JDM.

## Results

### JDM is associated with immunophenotypic differences in B and CD4^+^ T cell compartments.

**Figure 1:**
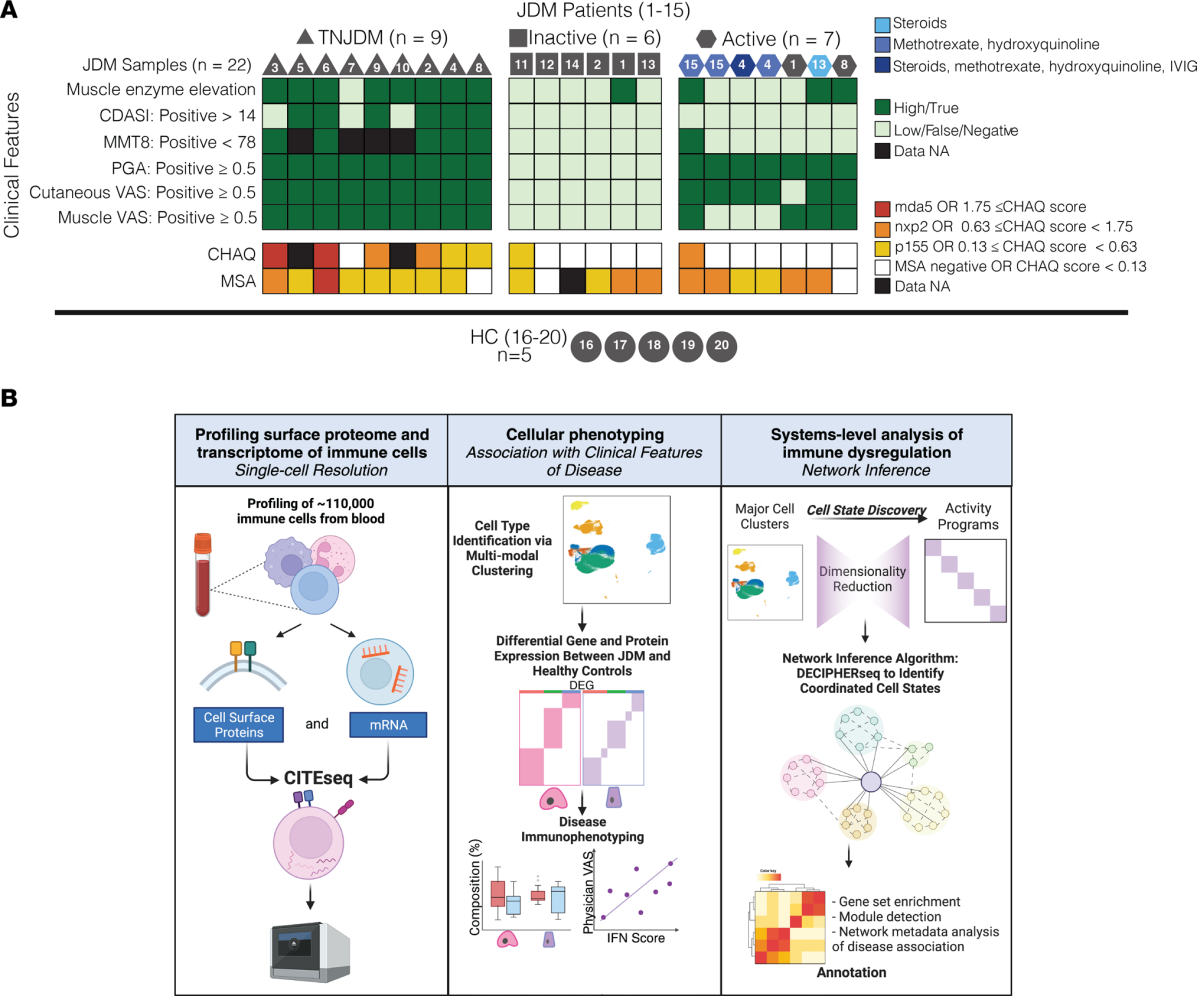
Study design and analysis strategy for profiling PBMCs from 27 samples. (**A**) Overview of clinical characteristics of study cohort. Individuals are labeled by donor ID (JDM 1–15, HC 16–20). Longitudinal samples were collected from the following donors: JDM1 (*n* = 2), JDM2 (*n* = 2), JDM4 (*n* = 3), JDM8 (*n* = 2), JDM13 (*n* = 2), JDM15 (*n* = 2). Icon shapes denote disease activity group, and shades of blue denote medication regimen. (**B**) Analysis strategy for CITEseq data from PBMCs. *n* = 22 JDM, *n* = 5 HC.

**Figure 2:**
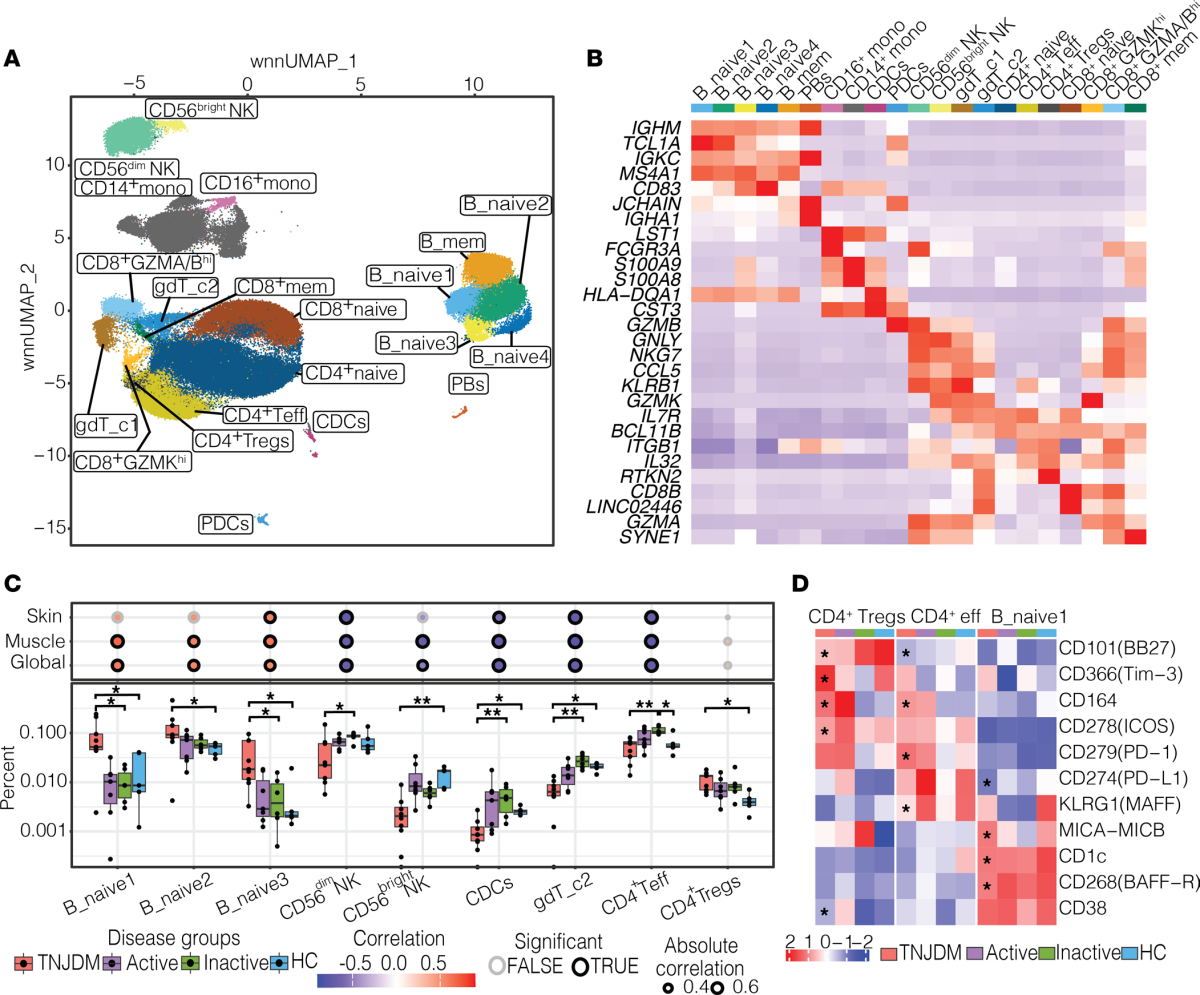
Cell types associated with JDM in peripheral blood. (**A**) UMAP constructed using weighted nearest neighbors (wnn) clustering colored by cell type. pDCs, plasmacytoid DCs; cDCs = classical DCs; PBs, plasmablasts; B_mem, memory B cells. (**B**) Heatmap with top 2 markers per cluster. (**C**) Box plot shows cell type proportion by disease group, using Kruskal-Wallis test with Dunn’s post hoc test comparing TNJDM with HC, TNJDM with inactive JDM, and inactive with HC (Holm’s, *P*_adj_ < 0.05; *P_adj_ < 0.05, **P_adj_ < 0.01). The dot plot above shows the Spearman correlation between corresponding cell type proportion in box plot and Physician Global VAS, where the size of the dot indicates the correlation, the color indicates the direction of the correlation, and the border weight indicates significance (*P*_adj_ < 0.05). (**D**) Heatmap with selected ADT protein markers. Asterisks mark significant comparisons between TNJDM and HC per cell type with an absolute LFC > 0.5 and *P*_adj_ < 0.05.

To gather a data set with appropriate controls and limited confounding, patients were selected according to disease activity and medication status (Figure 1A and Supplemental Table 1; supplemental material available online with this article; https://doi.org/10.1172/jci.insight.176963DS1). Of the 15 patients with JDM, serial samples were collected from 6 individuals, totaling 22 samples. Detailed information on sample numbers from patients can be found in Figure 1A. To minimize confounding by immune suppression, the study included 9 treatment-naive samples as well as 6 samples from patients with inactive disease off medication. CITEseq was performed on PBMCs to generate single-cell libraries (Figure 1B). Surface protein expression was measured using antibody-derived tags (ADT). Following preprocessing steps, we identified 29 clusters, which comprised 21 distinct immune cell populations across 105,827 cells (Figure 2A). Clusters were annotated using canonical RNA (Figure 2B) and protein markers (Supplemental Figure 1, A and B) within all major mononuclear immune cell compartments.

We first characterized global changes to cell composition across disease states comparing treatment naive JDM (TNJDM), inactive JDM, and HC (Figure 2C). Within the T cell compartment, the proportion of Tregs (CD45RO^+^, IL2R^+^, FOXP3*^+^*) was increased in patients with TNJDM (*P* = 0.02) consistent with previous findings (14). CD4^+^ effector T cells (CD45RO^+^) and γδ T cluster 2 (*TRDC*, *TRGC*) were significantly increased in patients with inactive JDM, and the proportion of cells from these populations negatively correlated with disease activity measures (*P* < 0.05). There was an overall decrease in innate populations in TNJDM compared with HC and inactive JDM, and the proportion of these cell types also correlated negatively with disease activity (*P* < 0.05).

Compared with HCs and patients with inactive disease, treatment-naive patients had higher proportions of multiple naive B cell populations, herein referred to as “B_naive,” and their UMAP cluster number, including B_naive1 (IgM^+^IgD^+^CD38^+^CD24^+^CD10^+^) corresponding to an immature naive B population, B_naive2 (IgM,^+^IgD^+^CD38^lo^CD24^lo^), and B_naive3 (IgM^+^IgD^+^CD38^+^CD24^+^), and the proportion of these populations positively correlated with multiple disease activity measures (*P* < 0.05) (Figure 2C). The proportion of B_mem cells, characterized by *TNFRSF13B* expression, negatively correlated with the muscle VAS score (*P* < 0.05). The immature naive B population had higher expression of CD38 (both RNA and protein) and *MZB1*, 2 genes essential for plasma cell differentiation, compared with all other B cell clusters (21, 22).

Given the observed imbalance of lymphocytes in treatment-naive JDM, we next sought to immunophenotype B cell and CD4^+^ T cell subsets in JDM at the proteomic level to gain molecular insight into cell states (Figure 2D and Supplemental Table 2). Differential protein analysis of immature naive B cells comparing treatment-naive JDM to HC identified increased expression of MICA-MICB and decreased expression of CD1C, BAFF-R, and PD-L1 (Figure 2D). Within the CD4^+^ T compartment, CD4^+^ Tregs from TNJDM had higher expression of Tim-3, ICOS, CD164, and CD38 and downregulation of CD101 a molecule which decreases proinflammatory T cell responses (23). CD4^+^ Teff in patients with TNJDM had higher surface expression of CD164 and PD-1 and showed downregulation of KLRG1, an inhibitory molecule (Figure 2D). The overexpression of PD-1 on the cell surface suggested that peripheral Th cells might be present in JDM (24, 25). However, while ICOS expression was higher (*P* < 0.05), no difference was found in surface expression of CXCR5 between CD45RO^hi^PD-1^hi^CD4^+^ T cells and CD45RO^lo^PD-1^lo^CD4^+^ T cells, and these cells were not significantly expanded in JDM (Supplemental Figure 2).

### SIGLEC-1 expression is a composite measure of the IFN gene signature in JDM.

We next compared gene and protein expression between treatment-naive JDM and HC samples in all cell types based on the hypothesis that certain cell types may not be altered in composition but may be functionally altered at the molecular level. Monocytes displayed the highest number of differentially expressed genes and proteins in this analysis, including CD169 (SIGLEC-1), CD107a (LAMP-1), and CD164 (Supplemental Figure 3). SIGLEC-1 is a monocyte-restricted IFN-induced protein that was recently identified as a potential biomarker in JDM (26). Both CD107a and CD164 are cell adhesion molecules involved in trafficking of activated PBMCs and adhesion to vascular endothelium (27).

**Figure 3:**
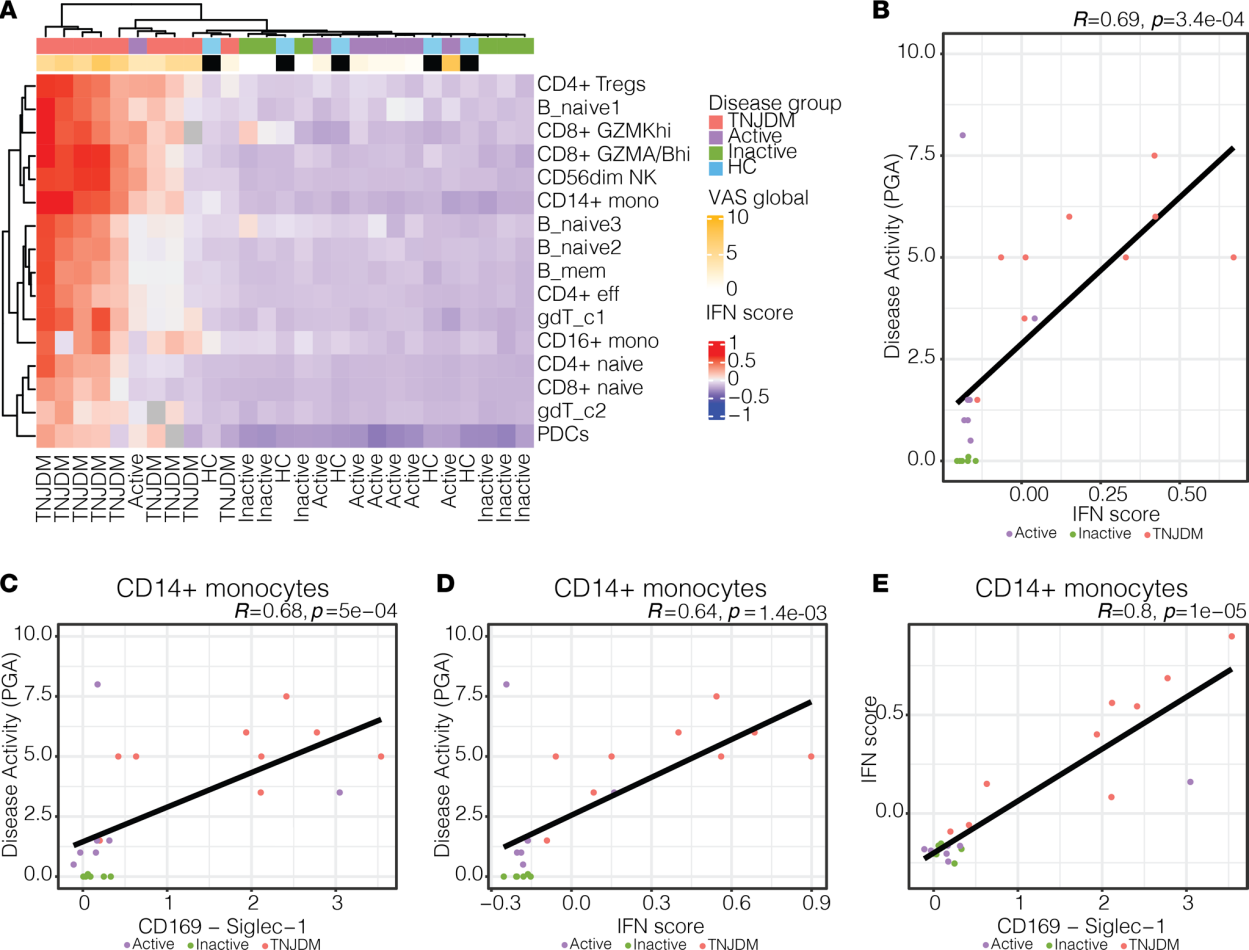
Type I IFN–induced gene and protein expression is associated with disease activity in JDM in CD14^+^ monocytes. (**A**) Heatmap of average IFN score per cell type and sample. Hierarchical clustering was performed using Euclidean distance and the complete clustering method. IFN score was calculated based on average expression of IFN module across all cells per sample. (**B**) Spearman correlation between IFN score and Physician Global VAS colored by disease group. (**C**) Scatter plot showing Spearman correlation between CD169 (SIGLEC-1) expression in CD14^+^ monocytes and Physician Global VAS. (**D**) Scatter plot showing Spearman correlation between IFN score and Physician Global VAS. (**E**) Scatter plot showing Spearman correlation between CD169 expression and IFN score in CD14^+^ monocytes.

A common finding across all cell types when comparing treatment-naive JDM and HC samples was overexpression of genes enriched in type I IFN processes, which was previously reported in bulk expression data and confirmed in single-cell studies (Supplemental Figure 4) (14, 19, 28). Using an IFN gene score derived from the transcriptional data (Supplemental Figure 5), we plotted the per-patient average score in each cell type (Figure 3A). This approach did not detect IFN gene expression to persist beyond the treatment-naive state, and 2 patients with TNJDM had negligible IFN gene signatures as quantified by this method. This heterogeneity of the IFN gene signature was partly explained by disease activity level (Figure 3B), as a bulk IFN gene score correlated with disease activity (*r* = 0.69). However, the remaining unexplained heterogeneity of this IFN score exemplifies a limitation of utilizing gene scores identified through pairwise comparisons between subsets of the data.

Given that SIGLEC-1 is a type I IFN–induced protein, we investigated whether patterns of type I IFN–stimulated gene expression were reflected at the protein level, as protein biomarkers are more amenable for clinical lab-based testing. SIGLEC-1 expression in CD14^+^ monocytes also correlated with disease activity to a similar degree as the IFN gene signature (Figure 3, C and D), and SIGLEC-1 expression was itself highly correlated with the IFN gene signature in monocytes (Figure 3E). This suggests that SIGLEC-1 expression in CD14^+^ monocytes is a representative composite measure of the IFN gene signature in JDM. These results underscore the potential of SIGLEC-1 as a biomarker of IFN responses in JDM that may be useful for tracking disease activity.

### Unsupervised network analysis reveals coordinated cell states shared among immune cells in JDM.

**Figure 4:**
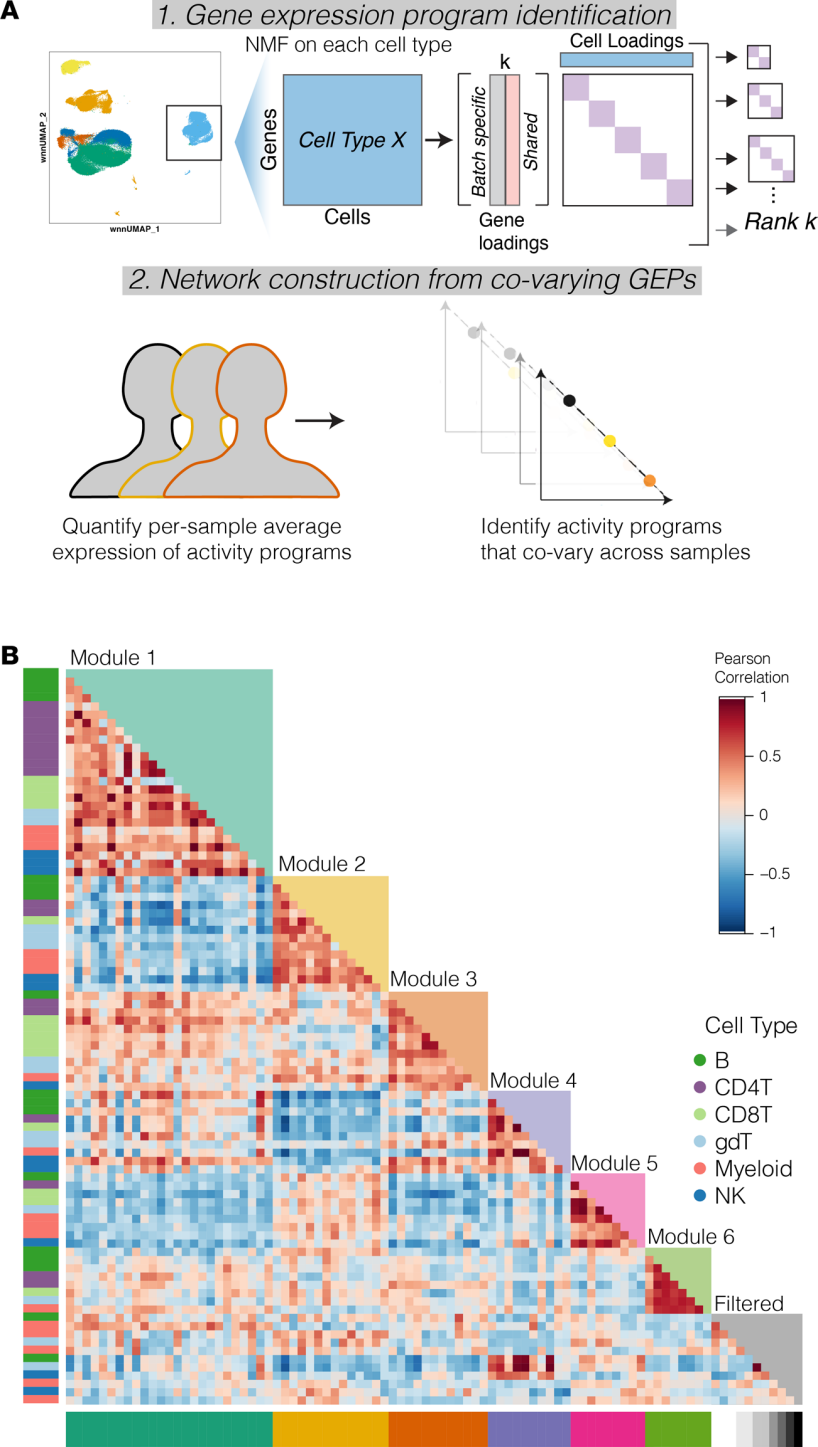
DECIPHERseq extracts gene expression programs from single-cell RNA-Seq data in JDM. (**A**) Overview of the DECIPHERseq workflow. (**B**) Heatmap showing 6 major clusters of GEPs identified by DECIPHERseq (Pearson). GEPs are clustered into modules, with isolated GEPs filtered out (grayscale).

We next turned to a systems-level approach to better understand the coordination of immune cell gene programs in JDM relative to HCs and in relation to disease activity level. We applied an unsupervised network inference method, DECIPHERseq, to the 6 major cell types annotated in the data set: B cells, CD4 T, CD8 T, NK cells, γδ T cells, and myeloid cells (Figure 4A). DECIPHERseq relies on nonnegative matrix factorization (NMF) to first break the data set down into gene sets that represent distinct states of biological activity, or “activity programs,” and then constructs a network of gene expression programs (GEPs) based on how expression of the programs covaries across patient samples (Figure 4A) (29). After outlier filtering, NMF identified 76 activity programs (Figure 4B).

**Figure 5:**
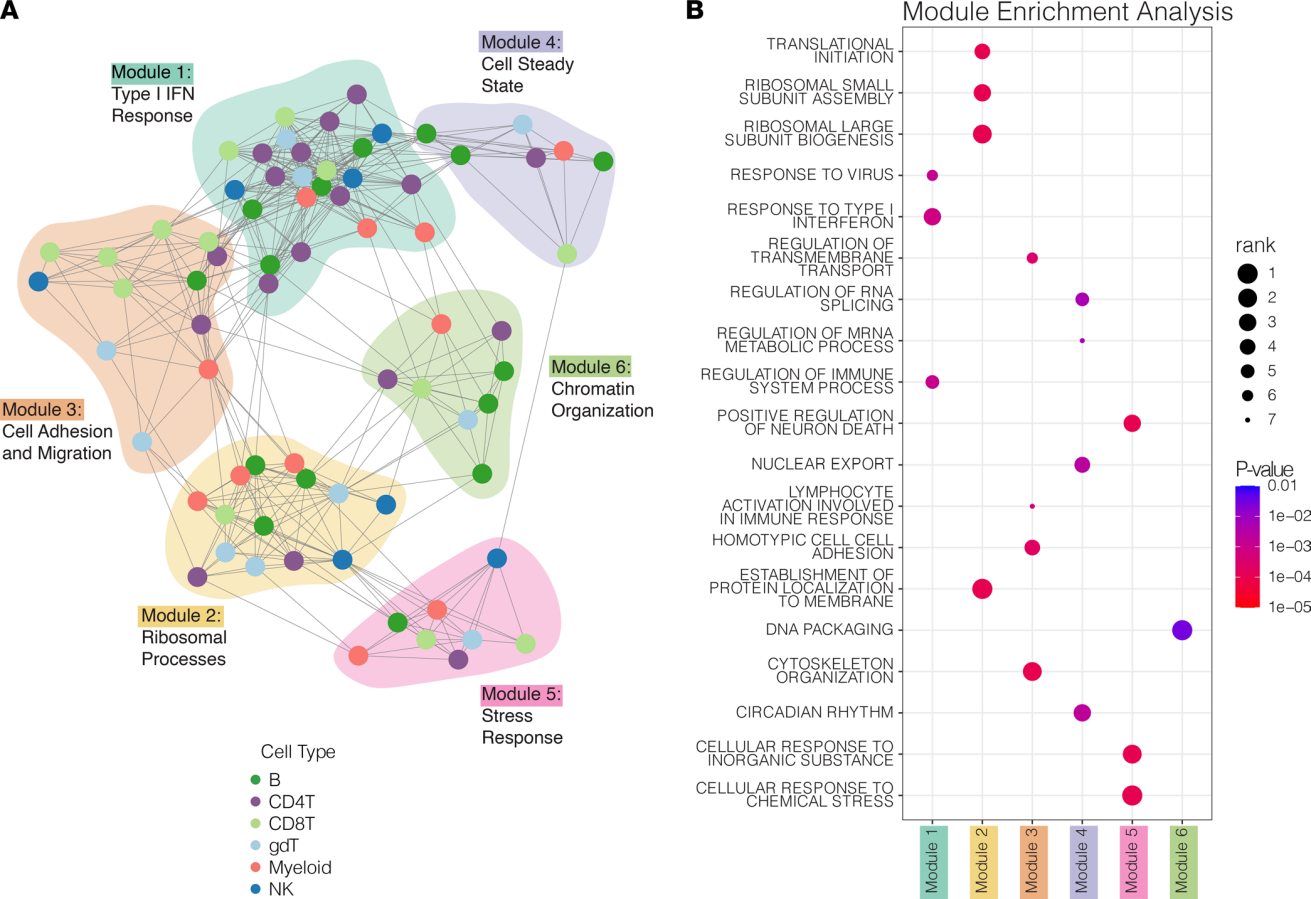
Network of coordinated biological activity inferred from GEPs in peripheral blood. (**A**) Network constructed from correlated GEPs in PBMCs from patients with JDM and HCs. Nodes represent programs in the given cell types, and edges represent positive significant correlations (Pearson, *P* < 0.05). (**B**) Dot plot showing selected gene sets found to be enriched within specific modules compared with the rest of the network. Color corresponds to module enrichment *P* value, and size corresponds to a set’s rank in the list of significantly enriched gene sets for that given module ordered by ascending module enrichment *P* value (network permutations, GSEA, FDR < 0.01). All gene sets shown fall in the top 10 terms for their respective modules (total gene sets: 626).

Next, a force-directed network graph from the correlation matrix of activity programs was constructed in which each node represents a program and each edge represents a statistically significant (*P* < 0.05) positive correlation between 2 nodes (Figure 5A). Using DECIPHERseq’s community detection algorithm, we identified 6 hubs of interconnected activity programs or “modules.” All modules contained multiple cell types, highlighting that many biological processes in JDM are coordinated across several immune cell types (Figure 4B and Figure 5A). We annotated each node using gene set enrichment analysis (GSEA) of Gene Ontology (GO) terms on each program’s ranked marker gene list (Supplemental Tables 3–4 and Supplemental Figures 6–11) (30, 31).

DECIPHERseq’s module enrichment analysis identified consensus biological themes for each module in an unsupervised manner (Figure 5B and Supplemental Table 5). Module 1 was enriched for type I IFN response programs such as Response to Virus. Module 2 was enriched in ribosomal processes including Translational Initiation. Module 3 included lymphocyte programs and was significantly enriched for cell adhesion and migration. Module 4 represented cells’ steady state processes as it was enriched for gene sets like Circadian Rhythm. Module 5 was annotated as a Stress Response module and enriched for Regulation of Cell Death and Cellular Response to Chemical Stress. Module 6 contained few unique gene sets and was enriched for programs intrinsic to eukaryotic cells like DNA Packaging.

### JDM CD4^+^ T cells and B cells display persistent alterations in gene expression in both active disease and remission.

**Figure 6:**
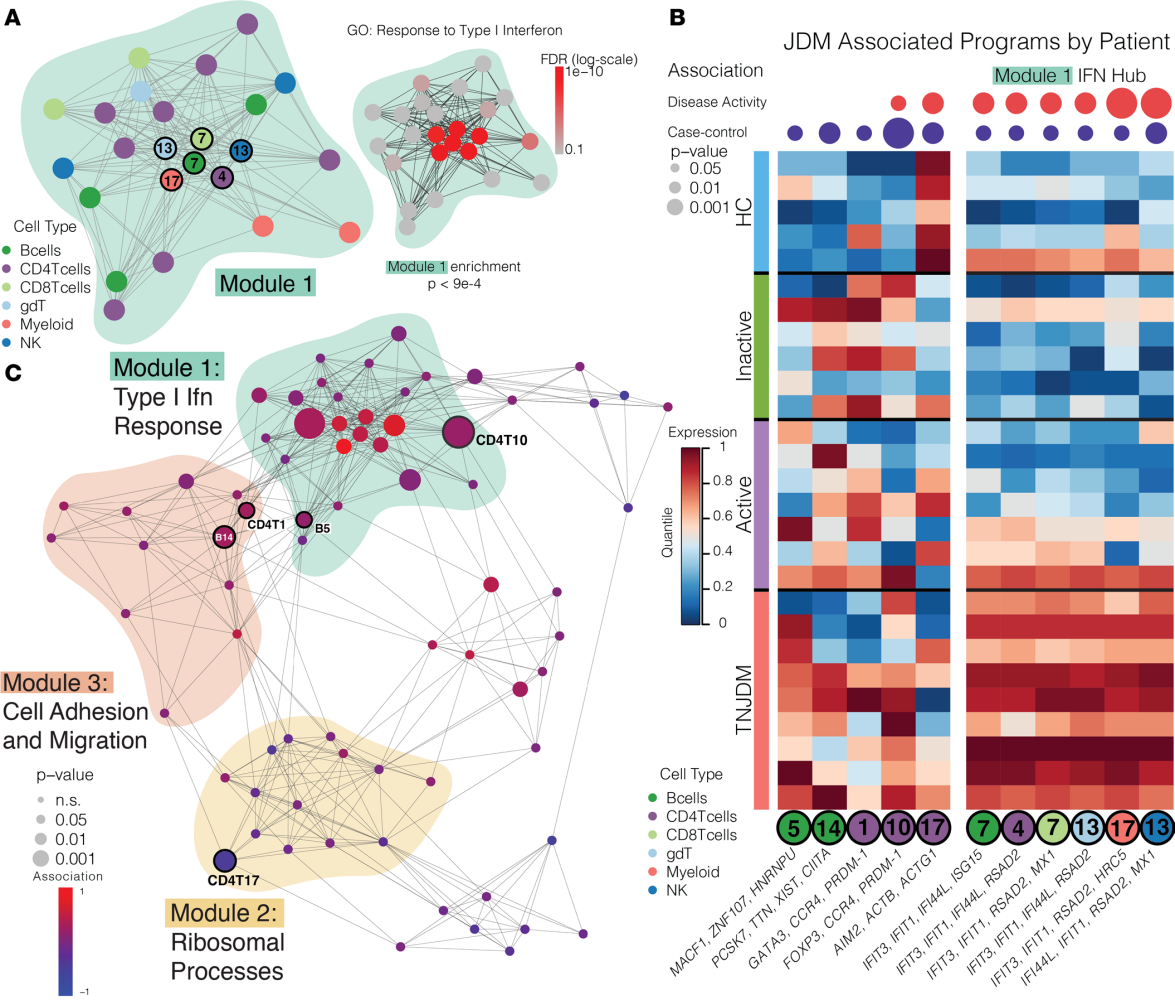
JDM is associated with a central IFN hub and cell-specific gene programs in the B and CD4 T compartments. (**A**) Zoomed-in graph of Module 1. GSEA results for Response to type I IFN GO term shown with each node colored according to FDR. *P*_adj_ value of module enrichment is also shown (network permutations, Methods). (**B**) Heatmap showing significant differences in expression of selected programs between HC (*n* = 5) and patients with JDM (*n* = 22), with columns annotated by *P* values (*P* < 0.05) of case-control (*t* test) and disease activity association (4-group 1-way ANOVA). (**C**) Network graph showing case-control analysis of each program’s expression, with node size scaled according to *P* value and colored according to strength of the association between disease status and program expression (*t* test).

Next, we aimed to interpret the annotated network in the context of GEPs associated with JDM compared with HCs irrespective of disease activity. We first focused on Module 1, which was enriched in type I IFN responses. Many programs in this module were increased in TNJDM, as expected (Figure 6, A and B). All 6 major cell types expressed IFN gene programs that were highly correlated to one another, as shown by the closely connected hub at the center of Module 1 (Figure 6A). This IFN hub was associated with JDM as compared with HCs (*P* < 0.05) (Figure 6, B and C). IFN modules identified by NMF were highly expressed in all treatment-naive patients as well as some patients with active disease, inactive disease, and a HC (Figure 6B), in contrast to the signature of IFN gene expression previously detected by differential gene expression in Figure 3A. This highlights the strength of this method to more accurately reflect the low-dimensional space of gene expression where measurement of many genes working together may be needed to detect underlying biological processes (32–34).

We next identified gene programs in Modules 1–3 expressed more highly in all patients with JDM compared with HCs (*P* < 0.05). These included B cell (5 and 14) and CD4T (1, 10, 17) programs, and their expression persisted even in patients with inactive JDM who previously achieved remission off medication (Figure 6B and Supplemental Figure 12). Patients with JDM more highly expressed 2 B cell programs; B5 in Module 1 was enriched in mRNA metabolic processing, RNA splicing, chromatin organization and modification, and cell cycle regulation, and B14 in Module 3 was enriched in chromatin remodeling and cytoskeletal organization (Supplemental Figure 6 and Supplemental Figure 8). These enriched biological processes suggest that a subpopulation of B cells are more transcriptionally active and undergoing epigenetic regulation in JDM relative to HCs.

In Module 3, correlated to B14, CD4T1 (enriched in cell migration, adhesion, activation, and secretion) was expressed more highly in JDM and in the region of the Uniform Manifold Approximation and Projection (UMAP) corresponding to CD4^+^ Teff (Supplemental Figure 13). This CD4T1 program expressed by CD4^+^ Teff contained genes (*GATA3*, *CCR4*, *PRDM1*) that indicate possible skewing toward a Th2 subset, while expression of *PRDM1* (Blimp-1) suggests participation in extrafollicular reactions (Figure 6B). Th2 CD4^+^ T cells were previously found to be expanded in JDM and associated with extrafollicular B cell–T cell help (15, 16). We observed similar expression of Th2 genes (*GATA3*, *CCR4*, *PRDM1*) in CD4T10, a Treg program (*FOXP3*, *IKZF2*, *IL2RA*) expressed more highly in JDM (Figure 6B). CD4T1 and CD4T10 included genes for costimulatory molecules OX40 (*TNFRSF4*) and GITR (*TNFRSF18*), both of which have been described to promote survival and proliferation of CD4^+^ Teff and have been targets of autoimmune disease therapeutics (Supplemental Table 4) (35–39). Notably, CD4T17 (*AIM2*, *ACTB*, *ACTG1*, *NCF1*, *ID3*, *SOX4*) was negatively associated with JDM, and expression was significantly decreased in nearly all patients (Figure 6B, Supplemental Table 4, and Supplemental Figure 14A). This program was enriched in protein targeting to the membrane and endoplasmic reticulum and included several genes important in T cell regulation. *NCF1* has been found to be a critical regulator of T cell tolerance in a collagen-induced arthritis mouse model (40), and coexpression of *ID3* and *SOX4* transcription factors has been identified as a mechanism of CAR-T cell exhaustion and dysfunction (41). Together, these results suggest multiple mechanisms by which CD4^+^ T cell dysfunction may occur in JDM, including participation in extrafollicular reactions, expression of costimulatory molecules, and downregulation of genes important in mediating tolerance and exhaustion.

### Immune cell states are correlated with IFN gene expression in treatment-naive JDM.

**Figure 7:**
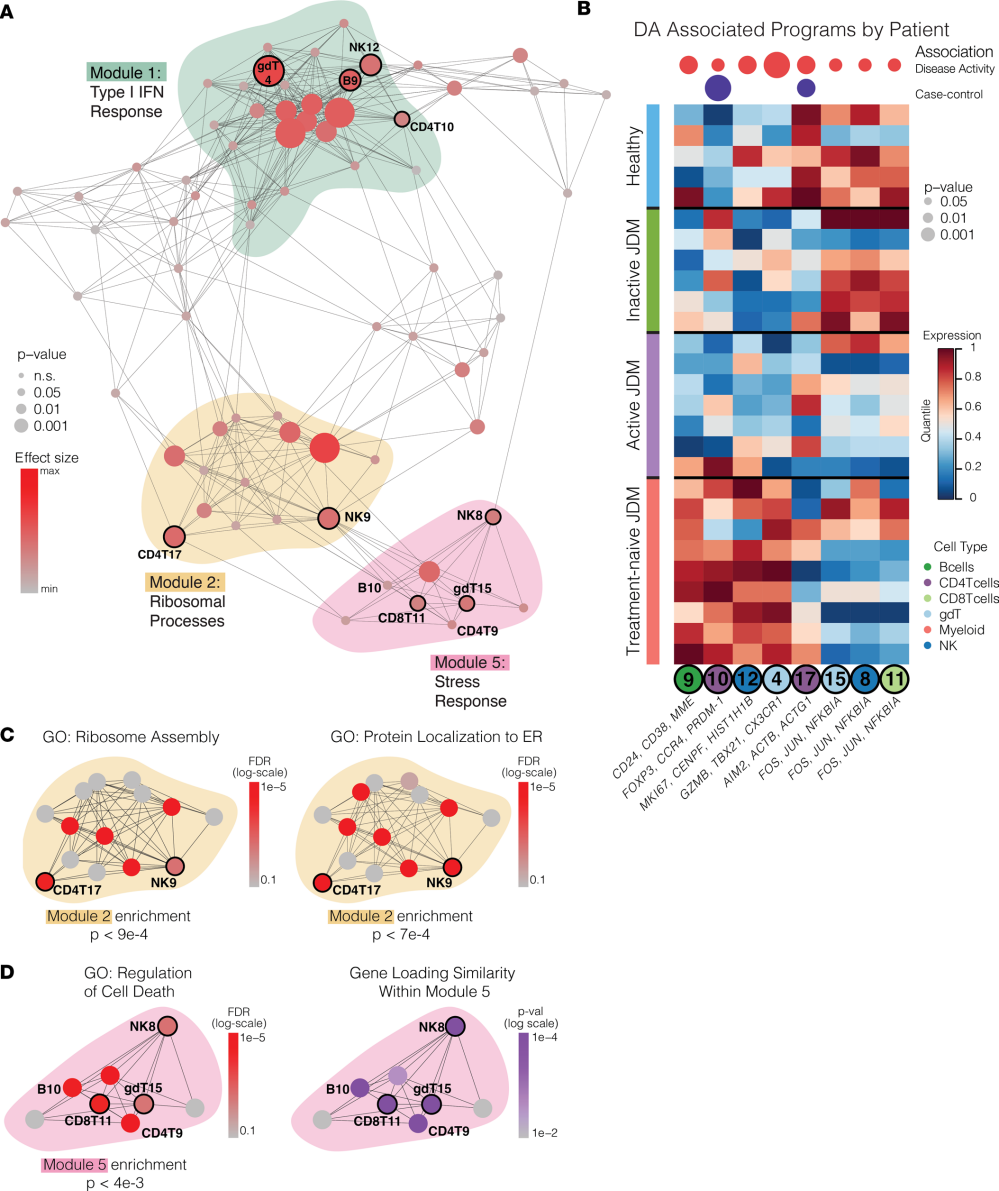
Disease activity in JDM is associated with central hub of IFN response in network, correlated with dysregulated immune cell states. (**A**) Network graph showing results of 4-group 1-way ANOVA of each program’s expression, with node size scaled according to *P* value and colored according to strength of the association between disease status and program expression. (**B**) Heatmap showing significant differences in expression of selected disease activity–associated programs between HC (*n* = 5) and patients with inactive JDM (*n* = 6), active JDM (*n* = 7), and TNJDM (*n* = 9). Columns are annotated by *P* values of case-control *t* test and disease activity association (4-group 1-way ANOVA). (**C** and **D**) Selected network modules colored by FDR of enrichment for indicated gene ontology set (FDR < 0.01) or gene loading similarity within Modules 2 (**C**) and 5 (**D**).

We next wanted to identify modules and gene programs associated with stages of disease activity in JDM (HC, inactive and active JDM, and TNJDM). To do so, we performed a 4-group 1-way ANOVA on each program in the network and post hoc pairwise analysis using the Tukey test. We identified programs in Module 1, 2, and 5 that were significantly associated with disease activity (*P* < 0.05) (Figure 7A). We confirmed that these biological programs moved in the direction expected within most patients with longitudinal assessments (Supplemental Figure 15). The IFN gene programs were also significantly overexpressed in treatment-naive patients with JDM, as expected (Figure 6B). Notably, expression of the central Module 1 IFN hub GEPs in all 6 major cell types more strongly correlated to the clinically evaluated PGA than the pseudobulk IFN gene score derived from pairwise DEG analysis (Supplemental Figure 16), underscoring the utility of a dimensionality reduction approach in uncovering clinically relevant gene signatures.

By isolating these IFN GEPs in each cell type, we were able to determine disease activity–associated programs correlated with the IFN hub, some of which corroborate previous findings (Figure 7, A and B). This approach identified B9, an immature naive B cell program (*CD24*, *CD38*, *MME*; Supplemental Table 4) to be significantly associated with disease activity (Figure 7B). This gene program shared several top markers (*TCL1A*, *SOX4*, *NEIL1*) with the immature B cell population that was previously found to be expanded in treatment-naive JDM (15, 23) (Supplemental Table 4 and Supplemental Figure 14B). Notably, expression of this activated immature B cell program could be attributed to the B_naive1 cluster that we observed to be increased in treatment-naive JDM during the compositional analysis (Supplemental Figure 17). Similarly correlated with the IFN hub, NK12 was associated with treatment-naive JDM compared with active and inactive disease (Figure 7B and Supplemental Figure 18A). NK12 (*MKI67*, *HIST1H1B*) was enriched for gene sets related to cell proliferation and epigenetic regulation, confirming findings that a subset of NK cells in JDM are highly activated and proliferative (Supplemental Table 4 and Supplemental Figure 14C) (13, 17).

We next focused our attention on the other disease activity–associated programs that DECIPHERseq identified as correlated with the Module 1 IFN hub. Importantly, these disease activity associations were only revealed in the lower dimensional space of gene sets identified by NMF rather than the noisier space of differential expression of individual genes. We annotated CD4T10, also significant in the case control analysis, as a proliferative Treg program (*FOXP3*, *IL2RA*, *MKI67*) which expressed genes implicated in extrafollicular B cell–T cell interactions (*PRDM1*) and genes associated with Th2-mediated inflammation (*GATA3*, *CCR4*; Figure 7B and Supplemental Table 4) (42). Notably, CD4T10 included the marker *CCR4*, a chemokine receptor highly expressed in Tregs that are preferentially recruited to skin under inflammatory conditions (43). Expression of both CD4T1 and CD4T10 colocalized with surface protein expression of CCR4 in the UMAP as well (Supplemental Figure 19), highlighting the advantage of this multimodal sequencing approach in identifying functional markers of transcriptomic signatures.

This network approach also identified the program γδ T4, a cytotoxic Th1 polarized γδ T program (*GZMB*, *CX3CR1*, *TBX21*) that was correlated with the central IFN hub and was significantly increased in treatment-naive patients compared with both active and inactive JDM and HC (*P* < 0.05; Figure 7B and Supplemental Figure 14D). High expression of *TRGC1* and *TBX21*, encoding the transcription factor T-bet responsible for regulating IFNG expression, specifically identified cells expressing this program as Th1-like TCRVd1 γδ T cells (Figure 7B and Supplemental Table 4) (44). A similar subpopulation of γδ T cells was found to be increased in synovial fluid and blood of patients with juvenile idiopathic arthritis, which expressed IFN-γ and TNF-α to the same degree as CD4^+^ T cells (45). This suggests this subpopulation of γδ T cells may reflect an important inflammatory cell state specific to treatment-naive disease correlated with IFN gene expression.

### Regulatory cell death and protein targeting pathways are dysregulated across multiple immune cell populations in JDM.

The disease activity programs that were highly expressed in treatment-naive JDM were components of Module 1, which was enriched for type I IFN and its associated immune processes. The network-wide 1-way ANOVA also revealed disease activity–associated programs in Module 2 and Module 5 (Figure 7A), which were anticorrelated with Module 1 (Supplemental Figure 20), and expression was decreased in treatment-naive patients with JDM compared with HCs and other patients with JDM (Figure 7B). Module 2 was significantly enriched for GO terms “ribosome assembly” and “translational initiation” (Figure 7C), while Module 5 was enriched for terms “regulation of cell death” and “cellular response to chemical stress” (module enrichment, *P* < 0.005) (Figure 7D). The disease-associated programs within these modules were expressed significantly lower in treatment-naive JDM, suggesting dysfunction of cellular processes that underpin ribosomal activity and cell death regulatory processes at disease onset (Figure 7B and Supplemental Figure 18, B–D).

Notably, disease activity–associated programs CD8T11, NK8, and γδ T15 (*FOS*, *JUN, DUSP1*, *NR4A2*, *GADD45B*) in Module 5 share a common gene signature (Figure 7B; Supplemental Figure 14, C and D; and Supplemental Table 4) and are each individually enriched in “regulation of cell death” and “regulation of cell cycle” (Figure 7D and Supplemental Figure 10). We quantified the overlap in gene expression between activity programs by Fisher’s exact test and confirmed the high gene loading similarity between programs in Stress Response Module 5 (Figure 7D). All 3 of these programs were expressed at lower levels in active and treatment-naive JDM and negatively correlated with activated disease–associated cell signatures identified in Module 1 (Supplemental Figure 18, B–D). The gene loading similarity analysis revealed that the programs CD4T9 and B10 also share top marker genes (*FOS*, *JUN*, *DUSP1*, *NR4A2*, *GADD45B*) (Figure 7D and Supplemental Table 4); however, these 2 programs were not associated with disease activity status. This suggests that regulatory mechanisms of cell death may be uniquely disrupted in circulating cytotoxic cell populations in patients with active disease.

In Module 2, CD4T17 and NK9 were enriched in several gene sets related to protein processing, such as protein targeting to the ER (Supplemental Figure 7A). Interestingly, the CD4T17 program was also characterized by high expression of several genes encoding members of the actin protein family (*ACTB*, *ACTG1*; Supplemental Table 4). Given the crucial role actin filaments play in antigen recognition during the formation of the immune synapse, dysfunction in components of that protein machinery could have substantial effects on the immune system. Among other disease activity–associated programs, differential expression of CD4T17 between HCs and patients with JDM persisted even in patients who achieved remission off medication (Figure 7B). Taken together, disease activity–associated programs in Modules 2 and 5 highlight shared cellular processes that may be underactive in JDM, providing what we believe to be novel insights into potential cellular mechanisms that accompany the known signature of overactive IFN-response in JDM.

### JDM-associated signatures identified by DECIPERseq validated in an independent data set.

We next investigated whether these JDM-associated signatures could be identified in an independent set of samples. Using DECIPHERseq’s marker quantification method, we subset the genes in each JDM-associated GEP that contributed the most to that program. With each gene list as input, we calculated a proxy GEP metric that quantified rank-based expression of each program as the enrichment of that subset of genes in each cell (46). This proxy NMF GEP metric recovered signatures identified by NMF in the original data set (Supplemental Figure 21).

**Figure 8:**
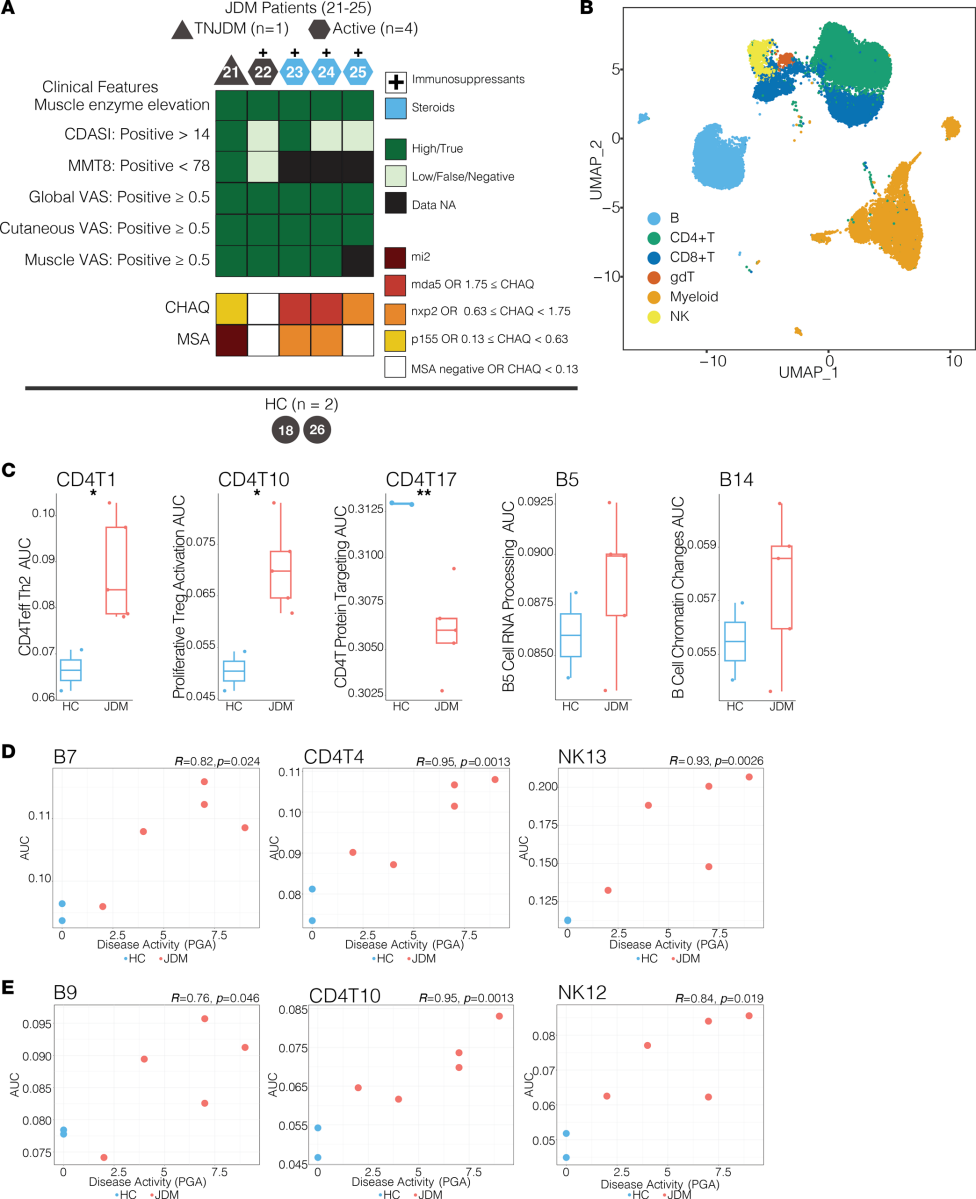
JDM-associated signatures identified by DECIPHERseq can be validated in independent samples. (**A**) Clinical characteristics of validation cohort (*n* = 7). HC18 was included in original cohort, but an independent sample was collected and analyzed for this data set. Individuals are labeled by the donor ID. Immunosuppressants denoted as “+” for patients JDM22–25 were as follows: (JDM22: methotrexate), (JDM23: IVIG, cytoxan), (JDM24: hydroxychloroquine, MMF, IVIG, tofacitinib), (JDM25: methotrexate, IVIG). (**B**) UMAP of single-cell RNAseq data from validation cohort PBMC samples, colored by 6 major cell types corresponding to labels used in original cohort. (**C**) Box plots of case-control comparisons (HC = 2, JDM = 5) for selected programs queried in validation data set using AUCell (*t* test, *P < 0.05, **P < 0.01). (**D** and **E**) Scatter plots correlating disease activity (PGA) with AUCell scores for selected IFN programs (**D**) and selected disease activity programs (**E**) in validation data set (Spearman, *P* < 0.05).

Using this proxy NMF GEP method, we validated key signatures in an independent set of CITEseq data from 5 JDM samples and 2 HC (Figure 8, A and B). Importantly, these samples were obtained from patients who had active disease, and 4 of 5 were being treated with medication. We compared GEP expression between cases and controls (*P* < 0.05), and we identified significantly higher expression of CD4T1 (CD4^+^ Teff program, *CCR4*, *PRDM-1*, *GATA3*) and CD4T10 (Treg program, *FOXP3*, *CCR4*, *PRDM-1*, *GATA3*) in JDM and lower expression of CD4T17 (*AIM2*, *ACTB*, *ACTG1*), replicating the original results in the initial cohort (Figure 8C). In the B cell compartment, B5 and B14 trended toward increased expression in patients with JDM. However, a single individual had low expression in both programs, indicating heterogeneity of expression of these B cell signatures (Figure 8C).

To validate disease activity–associated programs, we correlated GEP expression of disease activity–associated programs with the PGA score. It was infeasible to validate γδ T programs due to low cell numbers. IFN GEPs were most strongly correlated with disease activity in CD4T cells, B cells and NK cells (Figure 8D) and trended toward a positive correlation in myeloid cells, but there was no significant association in CD8 T cells (Supplemental Figure 22, A and B). Additionally, B9 (immature B cell signature; *CD38*, *CD24*, *MME*), CD4T10 (Treg program; *FOXP3*, *CCR4*, *PRDM-1*, *GATA3*), and NK12 (proliferative activated NK cell program; *MKI67*, *CENPF*, *HISTH1HB*) strongly correlated with disease activity (Figure 8E). A subset of the key signatures identified in the original cohort with DECIPHERseq trended with disease activity but did not significantly correlate with PGA in the validation cohort (Supplemental Figure 22, C–E). Together, these results demonstrate the robustness of many signatures quantified with a less precise method even in an independent data set with fewer samples. Cell death regulatory signatures previously identified to be negatively correlated with IFN signaling in cytotoxic populations may be more strongly associated with a treatment-naive state or more heterogeneous across healthy individuals such that significant differences in expression could not be identified in this smaller cohort.

## Discussion

Multiple components of the adaptive and innate immune compartments have been implicated in the pathogenesis of JDM, consistent with its categorization as a complex autoimmune condition. However, previous studies have been unable to uncover how multiple disease-associated cell states are coordinated to produce inflammation. Here, in the largest single-cell study of JDM to our knowledge to date, we provide an unbiased, comprehensive picture of immune dysregulation in peripheral blood, including a subset of aberrant signatures that persist despite disease quiescence in individuals off medication. Through traditional analyses, we first show that immune dysregulation in JDM manifests at the level of compositional imbalance of immune populations and that these compositional changes are correlated to clinical metrics of disease activity. Next, we identified distinct disease-associated molecular signatures of lymphocyte and myeloid subsets through multimodal differential analysis and demonstrate that, of these markers, surface expression of SIGLEC-1 in CD14^+^ monocytes is a composite metric of disease activity and reflects the type I IFN response in JDM (28). Using DECIPHERseq to deconvolve disease-associated programs beyond the broad type I IFN response, we uncovered CD4^+^ T cell states that persist in JDM despite disease remission coordinated with downregulated cell death processes in cytotoxic immune populations. Together, these findings generate more nuanced hypotheses for disease etiology.

Within the B cell compartment, we observed skewing toward an immature state in treatment-naive disease and observed the distinct transcriptomic and proteomic signature of immature naive B cells consistent with what we and others previously reported (14, 19). Given that autoantibodies are thought to play a role in disease pathogenesis, this skewing of the B cell compartment would seem counterintuitive. However, given recent findings emphasizing the importance of extrafollicular B cell differentiation pathways through which autoreactive “activated naive” B cells are precursors to antibody-secreting cells, we hypothesize that this skewing may be suggestive of extrafollicular reactions in JDM (24, 47). In fact, the expanded immature naive population had higher expression of CD38 and MZB1, genes important for plasma cell differentiation, than all other B cell clusters. The overall low expression of CD27 and CXCR5 across all B cells made it difficult to conclude whether this population matches the double-negative B cell population associated with SLE (47). However, recent immunophenotyping work in a large cohort of patients with JDM that found simultaneous expansion of CXCR5^–^ central memory B cells and Th2 cells provides support for further investigation into extrafollicular B cell–T cell help in JDM (15). Alternatively, this skewing could represent more mature B cells homing to tissues as has been described in antisynthetase syndrome (48, 49). Functional work to support or negate the extrafollicular pathway in JDM will be critical to determine whether this is a targetable pathway therapeutically.

Accompanying these immunophenotypic changes in B cells, we observed complementary dysregulation in the T cell compartment that lends further support to the hypothesis of extrafollicular interactions in JDM. In populations of peripheral blood FOXP3*^+^* Tregs and CD4^+^ effector T cells, we identify a shared disease-associated signature composed of genes suggestive of Th2 activation (*GATA3*, *CCR4*), involved in promotion of survival and proliferation (*GITR*, *OX40*), and associated with extrafollicular T cell responses (*PRDM1*). Notably, this signature persisted in disease remission in patients off medication. Likewise, we identify a CD4^+^ T signature, decreased in all patients with JDM regardless of medication status and disease activity, containing genes crucial for tolerance (*NFC1*) and regulation (*ID3*, *SOX4*). This cell state could represent an inflammatory signature or a compensatory mechanism of CD4^+^ T cells in more long-standing disease. These findings are consistent with previous work that identified skewing of CD4^+^ T cells toward a Th2 phenotype in JDM and showed in vitro that peripheral Th2 cells were efficient in helping B cells, including stimulating antibody production (16). A previous study also showed that tertiary lymphoid structures are present in muscle of new-onset JDM, further supporting a role for extrafollicular reactions in JDM (50). Future work using paired blood and tissue with spatial information to immunophenotype cells interacting within these structures would further justify investigating therapeutic strategies that prevent homing of CD4^+^ T and B cells to sites of inflammation in tissue.

While other studies have reported that Tregs in JDM have diminished suppressive capacity, raising the possibility of Treg exhaustion (51), the results in this study show that the expanded population of peripheral Tregs in blood are proliferative and activated (*MKI67*, *IL2RA*, *IRF4*), taking on an effector phenotype. Likewise, the shared signature with CD4^+^ Teff suggests that these Tregs are coopting the transcriptional machinery of effector T cells as has been described by others (52, 53). In this data set, JDM Tregs also upregulate transcriptomic and proteomic expression of CCR4 — paralleled by increased expression of CCR4 in CD4^+^ effector T cells — which is preferentially expressed in Tregs recruited to the skin (43). Thus, we speculate that this expanded population of Tregs in JDM could represent a peripheral response to site-specific Th2-mediated inflammation in disease-affected tissue. Alternatively, these Tregs could be functioning in a reparative manner at sites of tissue damage. Future functional studies of peripheral blood and tissue-specific Tregs, particularly investigation of the influence of type I IFN on Treg-suppressive capacity in JDM, would provide mechanistic insight on this population’s role in disease pathogenesis. The potential translational effect of investigating Treg dysfunction is corroborated by active development of multiple therapeutics targeting Tregs for autoimmune diseases (54).

More broadly, we show that unsupervised approaches such as DECIPHERseq can be used to consolidate disparate findings into a systems-level understanding of how interactions among cell states could manifest in disease. Here, our network analysis revealed that a module of hyperactivated IFN response across cell types is coordinated with dysfunction in ribosomal biogenesis, protein processing, and the regulation of cell death that is also shared across many cell types. This model contextualizes recent work that has identified ribosomal dysfunction in NK cells as a disease signature in JDM but also raises the possibility that defective translational machinery is not unique to that cell population (13, 17). Given that type I IFN directly promotes the activation and proliferation of NK cells (55, 56), we speculate that NK cells in JDM are unable to properly translate cytolytic protein machinery required for effector function in response to IFN signaling, potentially perpetuating the IFN response. Similarly, the shared program between CD8T, γδ T, and NK cells that describes regulation of cell death and cellular stress response suggests a common dysfunction across cytotoxic cell populations in JDM. Given the importance of cytotoxic cells in clearing cellular debris including autoantigenic neutrophil extracellular traps shown to be pathogenic in JDM, dysfunctional cytotoxic populations could result in accumulation of such debris, thereby triggering an autoimmune response mediated by lymphocytes (11).

Finally, the observation that type I IFN responses increase with clinical metrics of disease activity adds to the growing body of work suggesting that disease activity in JDM correlates with this transcriptional signature (57). However, given the time and cost, it remains infeasible to use transcriptomic sequencing as a lab-based clinical diagnostic tool. These data point to surface expression of SIGLEC-1 in monocytes as a composite measure of the IFN gene signature in JDM and disease activity. Together with a recent independent study of JDM, we provide external validation that SIGLEC-1 is a suitable biomarker for disease monitoring to pursue in larger immunophenotyping studies, given the lower cost and ease of implementing screening by flow cytometry (26). Importantly, we show that SIGLEC-1 directly reflects the IFN gene signature using paired gene and protein expression measurements, strengthening support for its use as a biomarker. Further study of this biomarker, and the role of SIGLEC-1 in disease, is an important step toward precision care of JDM.

These findings should be interpreted in the context of the study’s limitations. First, despite being the largest single-cell study in JDM to our knowledge to date, sample numbers are still limited such that the study lacks statistical power to quantify the contribution of MSA status to disease heterogeneity. Furthermore, a majority of patients in the treatment-naive group are TIF1γ^+^, which could introduce a bias to disease activity–related programs, though it remains unknown whether MSA status is associated with distinct biological mechanisms. Some patients in the “active” disease group had relatively low disease activity, which may have prevented us from identifying more associations with disease activity. Additionally, this study lacked data from matched JDM skin and muscle, which would have enabled insight into how dysregulated cell states in blood might influence local microenvironments in disease-affected tissue. Although profiling blood limits the mechanistic insight compared with skin or muscle, it is a more suitable sample type for biomarker discovery, particularly in a pediatric disease that requires longitudinal monitoring, and future comparison with tissue data will enable us to identify populations in peripheral blood with tissue correlates. Lastly, the DECIPHERseq algorithm relies on a k selection procedure to accurately decompose the data. As a dimensionality reduction technique, NMF is distinct from principal component analysis in that there is no single solution for the number of patterns or components into which the data are segmented. Therefore, it is necessary to optimize the parameter “rank K” such that the NMF results capture the relevant biology at an appropriate granularity. We addressed this limitation of NMF by using the phylogenetic clustering–based k-selection method described by Murrow et al., where the authors demonstrate that saturation of this metric reflects the appropriate granularity of biological programs such that results are robust across multiple choices of rank K (20).

In summary, using CITEseq to profile compositional and functional imbalance of peripheral blood immune cells and the relationship to disease activity, we provide a comprehensive map of the coordinated immune dysregulation underlying JDM. We identify persistent transcriptional changes in B and CD4^+^ T cells associated with JDM that persist even in patients in remission off medication and reveal cell states associated with the IFN signature that generate hypotheses for the role of extrafollicular interactions in disease pathogenesis, drawing parallels to other autoimmune diseases. Importantly, we believe these findings pose a new paradigm to how we approach JDM treatment. The dysregulation of processes simultaneously with hyperactivation of other cell states necessitates that we identify therapeutic strategies that restore balance to the dynamic interactions between immune populations rather than simply turning off a set of pathways. Taken together, our work sets the stage for improving clinical management of JDM by providing a foundation for systems-level inquiry into the cellular basis of this disease. More broadly, application of a similar analytical strategy could provide insight into the immunologic basis of other childhood-onset autoimmune diseases characterized by a type I IFN gene signature.

## Methods

### Sex as a biological variable.

This study contained samples from human males and females. Sex was not considered as a biological variable in downstream analyses.

### Study cohort and sample processing.

Patients were recruited to the Juvenile Myositis Precision Medicine Biorepository between 2018 and 2023 and underwent informed consent. The diagnosis of JDM was per clinician judgment; however, all patients included in this study met EULAR/ACR classification criteria for “definite” juvenile idiopathic inflammatory myopathy based on typical skin manifestations of either Gottron’s and or heliotrope rashes (58). This study was approved by the UCSF IRB. Clinical data were collected by study investigators and recorded in a secure REDCap database. Treatment-naive JDM was defined as a new diagnosis of JDM with no systemic immune suppressant use in the prior 4 weeks. Inactive JDM was defined as normal CK, MMT8 ≥ 78 and Physician Global VAS score < 0.5 to reflect PRINTO clinically inactive disease (59) definitions but with some modifications based on the data available. Active disease was defined as Physician Global VAS score ≥ 0.5, and all patients in this category were taking immune suppressive medications. Longitudinal samples from *n* = 6 patients with JDM were included separated by at least 4 months in time and accompanied by a change in disease activity. Measures of disease activity, including the Cutaneous Disease Area and Severity Index (CDASI), were collected at study visits (60). HCs were enrolled who had no prior autoimmunity; no known or suspected genetic disorders, immunodeficiency, active cancer, or history of organ or bone marrow transplantation; no infection or antibiotics in the prior 4 weeks; no chronic systemic immunomodulatory medication use; and no vaccinations in the prior 6 weeks. Peripheral blood samples were collected at each study visit and processed by the Pediatric Clinical Research Core Sample Processing Lab (UCSF). PBMCs were collected in SepMate tubes (STEMCell, 85415) (*n* = 9) using Ficoll separation or CPT tubes (*n* = 18), isolated per manufacturer’s guidelines, and cryopreserved in liquid nitrogen.

### CITEseq of human PBMCs.

The experimental protocol was previously published (14). These experiments were carried out using early access kits from BD Genomics before the implementation of commercially available single-cell protein/RNA assays (e.g., Feature Barcoding, 10X Genomics; BD Abseq, BD Genomics; Supplemental Table 6), and researchers are recommended to use those newer solutions for any follow-up studies as the techniques and reagents have been refined. PBMCs from 27 distinct samples were gently thawed in a 37°C water bath and resuspended using a pipette set to 1 mL. Cell counts and viability were determined using a Cellometer Vision (Nexcelcom) with AOPI staining (Nexcelcom, CS2-0106-5ML). Cells were multiplexed into 4 pools: 1 “cross pool” with all samples that consisted of only 1 time point and 3 pools consisting of longitudinal samples. Longitudinal samples from the same individual were assigned to separate pools to enable genetic demultiplexing. After pooling, cells were resuspended in 90 μL of 1% BSA in PBS and Fc blocked with 10 μL Human Trustain FcX (BioLegend, 422302) for 10 minutes on ice before being stained on ice for 45 minutes with a pool of 268 antibodies in 100 μL, for a final staining volume of 200 μL. Antibodies were pooled on ice with 2.2 μL per antibody per 1 × 10^6^ cells (BD Genomics). Cells were quenched with 2 mL 1% BSA in PBS and spun at 350*g* for 5 minutes and further washed 2 more times with 2 mL of 1% BSA in PBS. After the final wash, cells were resuspended in 100 μL and strained through a 40 μM filter (SP Bel-Art, H13680-0040). Each longitudinal pool was split across two 10X Chromium lanes while the “cross pool” was split across six 10X lanes (6 wells total, 5 × 10^5^ cells/well). The 10X Chromium controller (10X Genomics, 1000202) was run, and post-GEM reverse transcription (RT) and cleanup were done according to manufacturer’s protocol (10X Genomics 3′ Kit V3). Starting at cDNA amplification, modifications to the protocol were made: 1 μL of 2 μM additive primer (BD Genomics, beta kit) specific to the antibodies tags was added to the amplification mixture. During the 0.6X SPRIselect (Beckman Coulter, B23318) isolation (0.6X ratio of SPRIselect beads/sample reaction volume) of the post-cDNA amplification reaction cleanup, the supernatant fraction was retained for ADT library generation. Subsequent library preparation of the cDNA SPRIselect pellet was done exactly according to protocol, using unique SI PCR Primers (10X Genomics). For the ADT supernatant fraction, a 1.8X (beads/original sample reaction volume) SPRIselect was done to isolate ADTs from other nonspecifically amplified sequences, followed by sample index PCR. Sample index PCR for the ADTs was done using the cycling conditions as outlined in the standard protocol (15 cycles) but using unique SI-PCR primers such that all libraries could be mixed and sequenced together. Subsequent SPRI selection was performed, and all libraries were quantified and analyzed via Qubit 2.0 (Thermo Fisher Scientific) and Bioanalyzer (Agilent), respectively, for quality control. We sequenced the libraries on 2 lanes of a NovaSeq S4 (Illumina), aligned using CellRanger (10X Genomics) to generate feature barcode matrices.

### Sequencing data preprocessing and integration.

Data were demultiplexed using genotypes with demuxlet (61), and doublets were filtered using DoubletFinder (62). Next, cells were filtered to remove RNA transcripts expressed in < 3 cells, cells with > 60% ribosomal reads and > 15% mitochondrial reads (mtDNA). Cells were further filtered for > 5,000 ADT counts to avoid antibody aggregates, < 70 antibodies detected, and any antibody isotype control measurements > 50. To remove background ambient RNA signal, we ran SoupX separately on each of the 6 RNA libraries before merging (63). Aggregated data were log-normalized and scaled, regressing out percent mtDNA, percent ribosomal DNA, and cell cycle (S, G2M) (64). Data were then integrated with Harmony, with 20 maximum iterations of Harmony and 30 maximum clustering iterations per round of Harmony (65).

Normalization was performed using the dsb algorithm on all 6 ADT libraries individually, using default parameters except for more stringent quantile clipping (lower threshold = 1st percentile, upper threshold = 99th percentile) (66). The background distribution of empty droplets was defined as suggested in the dsb vignette. Isotype controls were then removed from the data set, and reciprocal PCA (RPCA) was used to integrate the dsb-normalized ADT data across libraries. Following RPCA, the data were rescaled and the cell cycle scores and number of ADT counts and features were regressed out. The harmonized RNA and RPCA-corrected ADT were combined using weighted nearest neighbors, with default parameters except for the threshold for filtering edges in the Shared Nearest Neighbors (SNN) graph (‘prune.SNN’ = 1/20). Leiden clustering was run on the resulting graph (method = igraph) at a 1.4 resolution (67). Two clusters were removed with low to no expression of ADT, and the object was reclustered with the same parameters. The Seurat function FindAllMarkers was used to identify the top 5 markers per cluster.

We removed 3 additional clusters: 2 were small clusters with a transcriptomic profile consistent with doublets (original Leiden clusters 26 and 29; Supplemental Figure 23), and 1 was a diffusely expressed cluster (original Leiden cluster 19; Supplemental Figure 23). We further subclustered 3 clusters that expressed genes representative of more than 1 cell type: original Leiden clusters 16, 17, and 23. Subclustering was performed using Seurat’s FindSubCluster function using the lowest possible resolution to divide the population into 2 clusters. Based on minimal transcriptional differences between them, original Leiden clusters 1, 5, 9, 11, and 15 were merged into a single CD4^+^ T naive population; clusters 3 and 10 were merged into a single naive CD8^+^ T population; and cluster 7 and part of the subsetted cluster 23 were merged into a CD56^dim^ NK population. Due to interpersonal heterogeneity in monocytes, all CD14^+^ monocyte clusters were merged into a single CD14^+^ monocyte population (68).

While annotating, we discovered that the FOXP3 signature normally attributed to Tregs was only present in a subset of the cluster, and FindSubCluster did not appropriately isolate the FOXP3*^+^* cells. We therefore subsetted the cluster and reran FindVariableFeatures, ScaleData, RunPCA, FindNeighbours, FindClusters with the Louvain algorithm and a resolution of 0.8, and RunUMAP. This enabled us to subset a smaller group of cells with a statistically significant expression of *FOXP3* compared with other clusters using FindAllMarkers, which we hence annotated Tregs. Annotation was performed using both canonical gene and protein markers. One B cell population consisted almost solely of cells from 2 donors. This was annotated as B_naive4 and was not used in downstream analysis, but it was included in UMAPs.

### Cell type proportion analysis.

Cell type proportion was calculated as the proportion of each cell type for each individual and was compared for: treatment-naive JDM compared with HC, treatment-naive JDM compared with inactive JDM, and inactive JDM compared with HC using Kruskal-Wallis test with Dunn’s post hoc test. To determine the association between cell abundance and disease activity, the Spearman correlation coefficient between cell type proportion and physician global VAS scores was calculated, and *P* values were adjusted using Benjamini-Hochberg (BH).

### Differential gene and protein expression analysis.

The DGE and DPE analysis were completed using DESeq2. Size factors were set using the function computeSumFactors from the scran package. The DESeq function was used with default parameters for single cell data (69). Batch was included as a covariate using the “reduced” argument. We filtered genes and proteins that were not expressed in at least 5% of cells and analyzed only cell types where there were at least 100 cells in each group. We used cutoffs of |LFC| ≥ 1 for genes, |LFC| ≥ 0.5 for proteins, and *P* < 0.05. Overrepresentation analysis was performed on up- and downregulated genes per cell type using the clusterProfiler package with GO Biological Processes terms set as the reference and adjusted *P* < 0.05. For the PD1/CD45R0 subanalysis (70), we compared groups using Seurat’s FindMarkers with test.use = ‘MAST’, latent.vars = ‘well’, |LFC| ≥ 0.5, and *P* < 0.05.

### Identification of global IFN signature.

We curated a list of all genes differentially expressed between treatment-naive JDM and HC in at least 2 cell types. The average expression of each gene was calculated using Seurat’s AverageExpression function per sample for each cell type and visualized using dittoSeq’s dittoHeatmap (71) with default, unsupervised clustering settings of both rows and columns. Dendrograms were ordered using the dendsort package (72). This identified 7 distinct modules, where Module 1 consisted exclusively of type I IFN–stimulated genes. Average Module 1 scores for each cell type were then calculated using Seurat’s AddModuleScore with default settings. Correlations between disease activity and IFN score was calculated using Spearman correlation and visualized using ggplot2 (73).

### Network inference from RNA data using DECIPHERseq.

We applied NMF to the raw RNA count data as implemented in the DECIPHERseq method with default parameters and the NMF rank, k, chosen using the weighted subtrees metric based on phylogenetic clustering (20). The final choices of rank for each cell type were k_B_ = 17, k_CD4T_ = 17, k_CD8T_ = 14, k_γδT_ = 13, k_Myeloid_ = 17, k_NK_ = 11 according to the saturation point in the elbow plots (Supplemental Figure 24). Network clustering was performed on the per-sample averaged program scores with default parameters. Marker gene scores were calculated on the corresponding gene loading vectors for each GEP as previously described (74). GSEA was performed on the resultant ranked gene lists using the fgsea (75) package in R with GO and Hallmark gene sets. Module themes were assigned by calculating module enrichment *P* values using the Get_enrichment_pvals function in DECIPHERseq with default parameters. Module and gene set enrichment results were visualized using ClusterProfiler (76).

### Validating signatures in independent data.

We performed CITEseq using the same protocol as described above with PBMCs from 5 patients with JDM and 2 healthy pediatric controls. We used the same steps for data processing, with the exception that we used CellBender rather than SoupX for ambient RNA removal and clustered cells using the RNA measurements only (77). To derive proxy scores for GEP in the independent data set, we ranked the gene lists comprising each program by marker score, which quantifies how strongly a single gene contributes to that GEP. Using the top 5% of genes by marker score, that list was used as input for the rank-based gene subset enrichment method AUCell (78). Pseudobulk proxy GEP scores were calculated as the per-patient mean expression in the same way for the original NMF programs. Case versus control comparisons were done using 2-tailed *t* tests between patients with JDM and HCs. Given the patients per group (HC, 2; active JDM, 4; TNJDM, 1) in this validation cohort, we could not repeat the 1-way ANOVA comparisons used for disease activity association in the original data set. Instead, proxy GEP scores were correlated to VAS global using the Spearman correlation.

### Statistics.

All statistical analyses and visualization of results were performed using open-sourced R (version 4.2.3). Pairwise comparisons of cell proportions between patient groups were performed using a Kruskal-Wallis test with 2-tailed post hoc Dunn’s comparison, with *P* values adjusted for multiple comparisons by Holm’s correction. Significance of Pearson correlations between GEPs used for network construction was calculated using bootstrapping as implemented in DECIPHERseq. Analyses of disease association with GEPs was performed using 2-tailed unpaired *t* test or 1-way ANOVA with post hoc Tukey test. FDRs for GSEA annotation and module enrichment across programs were calculated and corrected at the cutoff FDR < 0.01. Gene loading similarity was calculated as the Pearson correlation between gene loadings for each activity program and all other activity programs in the same module, with *P* values calculated by permutation testing. Data are shown as mean ± SD. Correlation methods used in specific figures are described in the corresponding legends and in Methods, and significance for all statistical tests was set at the threshold *P* < 0.05.

### Study approval.

This study was approved by the UCSF IRB 17-24003. Written informed consent to participate in this study was provided by the participant or the participant’s legal guardian depending on the age of the participant. Assent was obtained when appropriate.

### Data availability.

The data sets presented in this study are deposited in the CZ CELLxGENE Discover resource as “CITEseq of JDM PBMCs” (https://cellxgene.cziscience.com/collections/c672834e-c3e3-49cb-81a5-4c844be4a975). The code used for this analysis has been made publicly available on Github at “github.com/grabadam-cal/jdm-DECIPHER-2024”. Values for all data points in graphs are reported in the Supporting Data Values file.

## Author contributions

GR and CW were assigned co–first authorship because their contributions were deemed equally essential to the study, with GR listed first because she led the writing of the manuscript. JN, SK, MS, and CJY designed the experiments. JN and SK recruited patients for the study. GCH, YS, and JN performed the experiments. GCH performed the alignment and sample demultiplexing. EF, GR, and CW performed the integration and quality control. CW performed the annotations and the cell proportion and differential analysis. GR conceptualized and wrote the pipeline for the network analyses; EF ran the pipeline. GR, CM, and GKF performed analysis of the second data set. ZJG, MS, and JN provided essential feedback on the analysis strategy and statistical methods. GR and JN wrote the manuscript; MS and ZJG revised the manuscript. JN, MS, and SK conceptualized the study. JN led the project administration and acquired funding for the study. All authors reviewed and edited the manuscript.

## Supplementary Material

Supplemental data

Supplemental table 1

Supplemental table 2

Supplemental table 3

Supplemental table 4

Supplemental table 5

Supplemental table 6

Supporting data values

## Version 1 05/14/2024

In-Press Preview

## Version 2 06/24/2024

Electronic publication
