# Supplementary material for: Coordinated immune dysregulation in juvenile dermatomyositis revealed by single-cell genomics
Source: JCI Insight. 2024 May 14;9(12):e176963. doi: 10.1172/jci.insight.176963 (PMC11383589; doi:10.1172/jci.insight.176963)
Supplement: Supplemental data [file jciinsight-9-176963-s178.pdf]

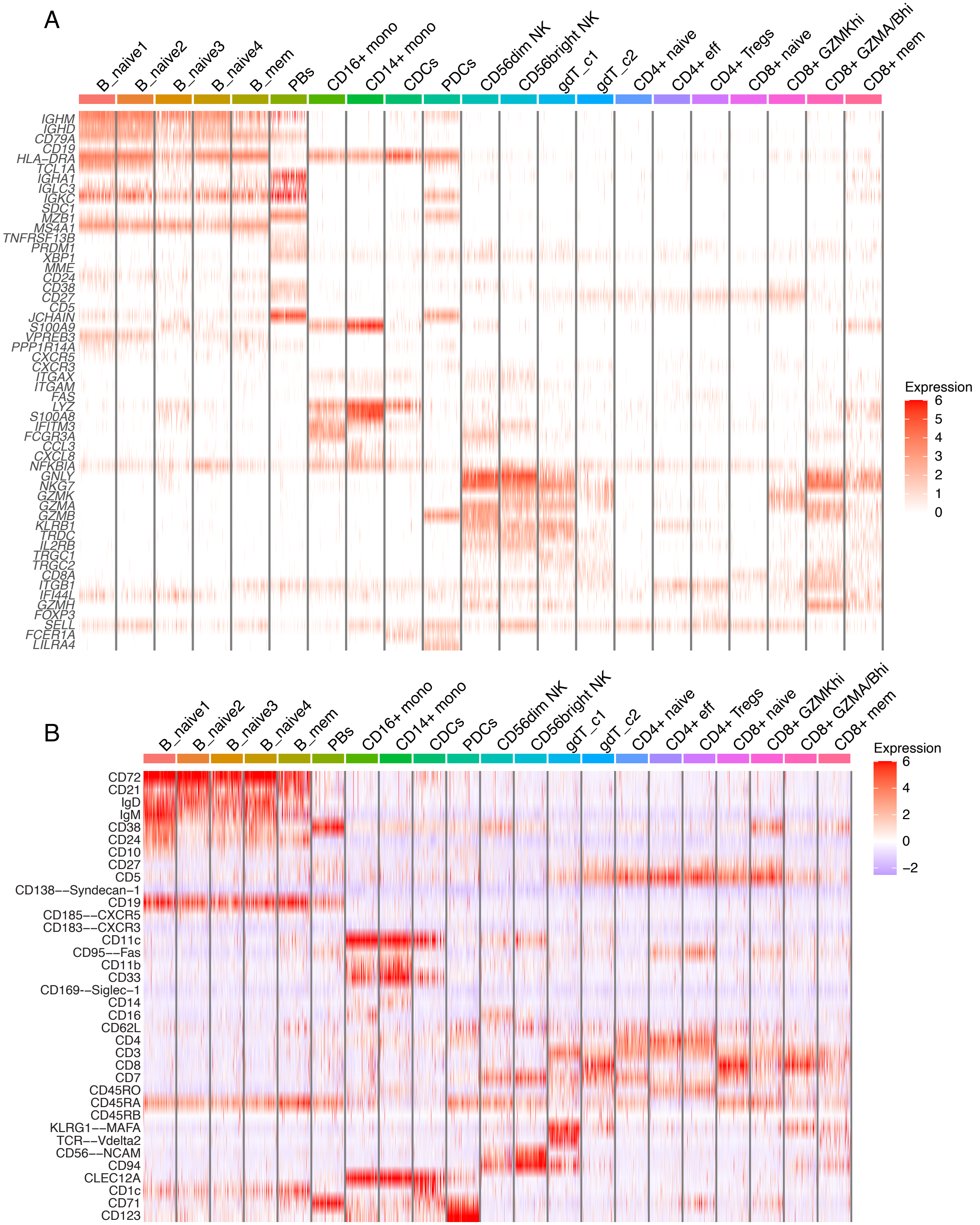

Supplemental Figure 1. Canonical RNA (A) and surface protein (B) markers for clusters in wnnUMAP shown in Figure 2.

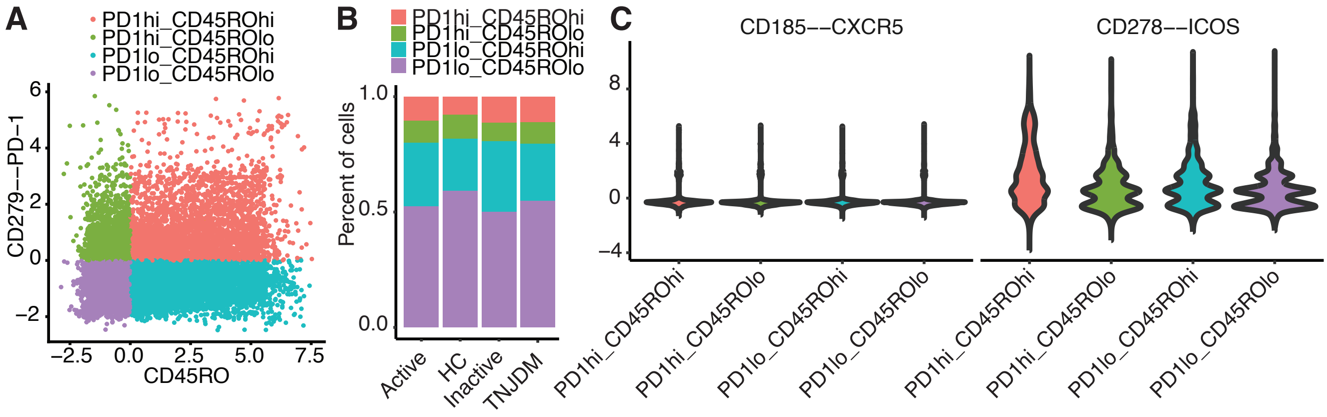

Supplemental Figure 2. (A) Dot plot of CD4+ T cells showing expression of CD45RO and PD-1. **(B)** Bar plot showing percentages of PD1/CD45RO-expression groups per disease group. **(C)** Violin plots shoring expression of CXCR5 and ICOS per PD1/CD45RO-expression group.

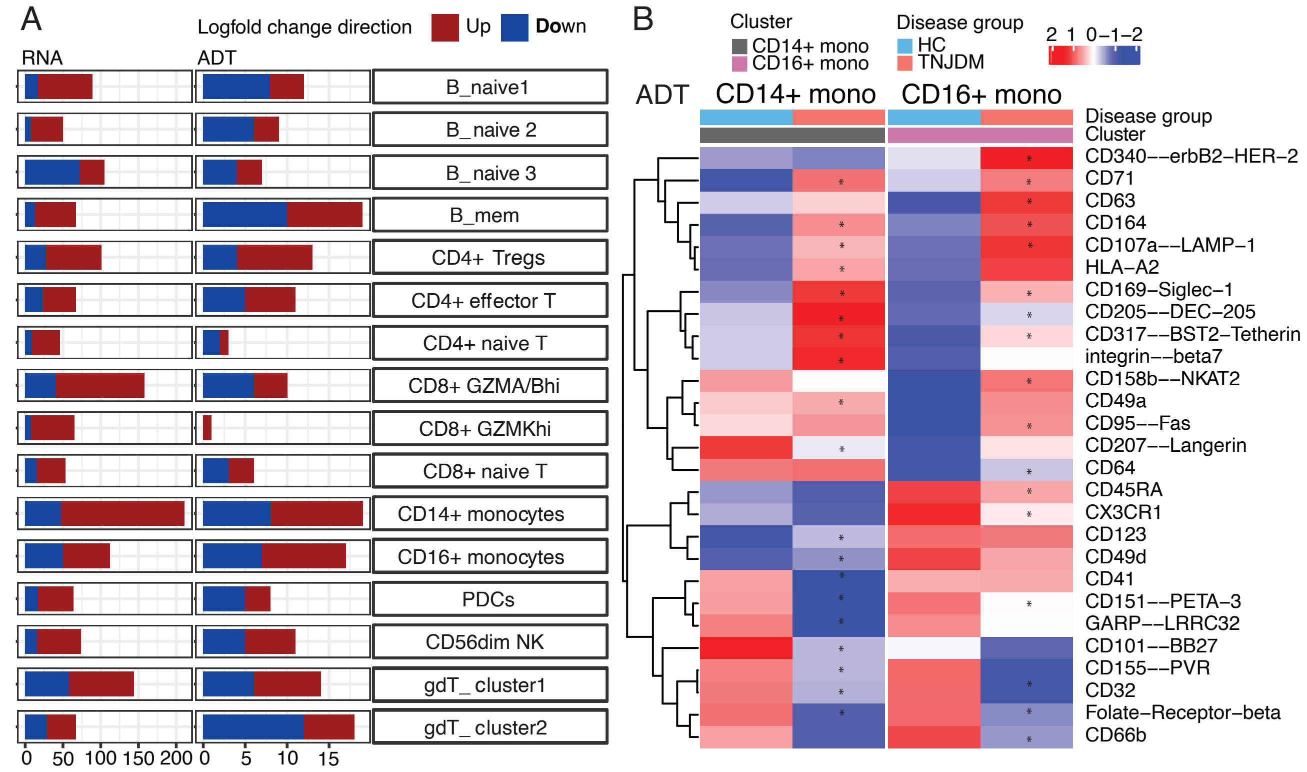

Supplemental Figure 3. (A) Differential analysis between treatment-naive JDM and HCs for each cell type. **(B)** Differential analysis of surface proteins. (TNJDM vs. HC) for monocytes. Asterisks indicate significant differential expression (DESeq2, BH adjusted $p < 0.05$).

A

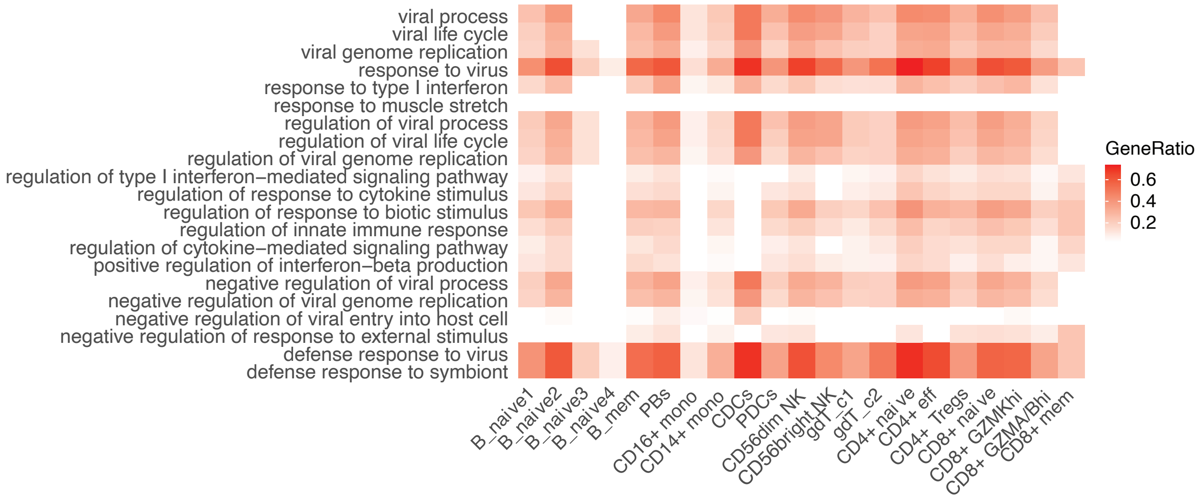

Supplemental Figure 4. Heatmap of top five enriched GO terms in all up-regulated genes per cell type from GOA with FDR<0.01.

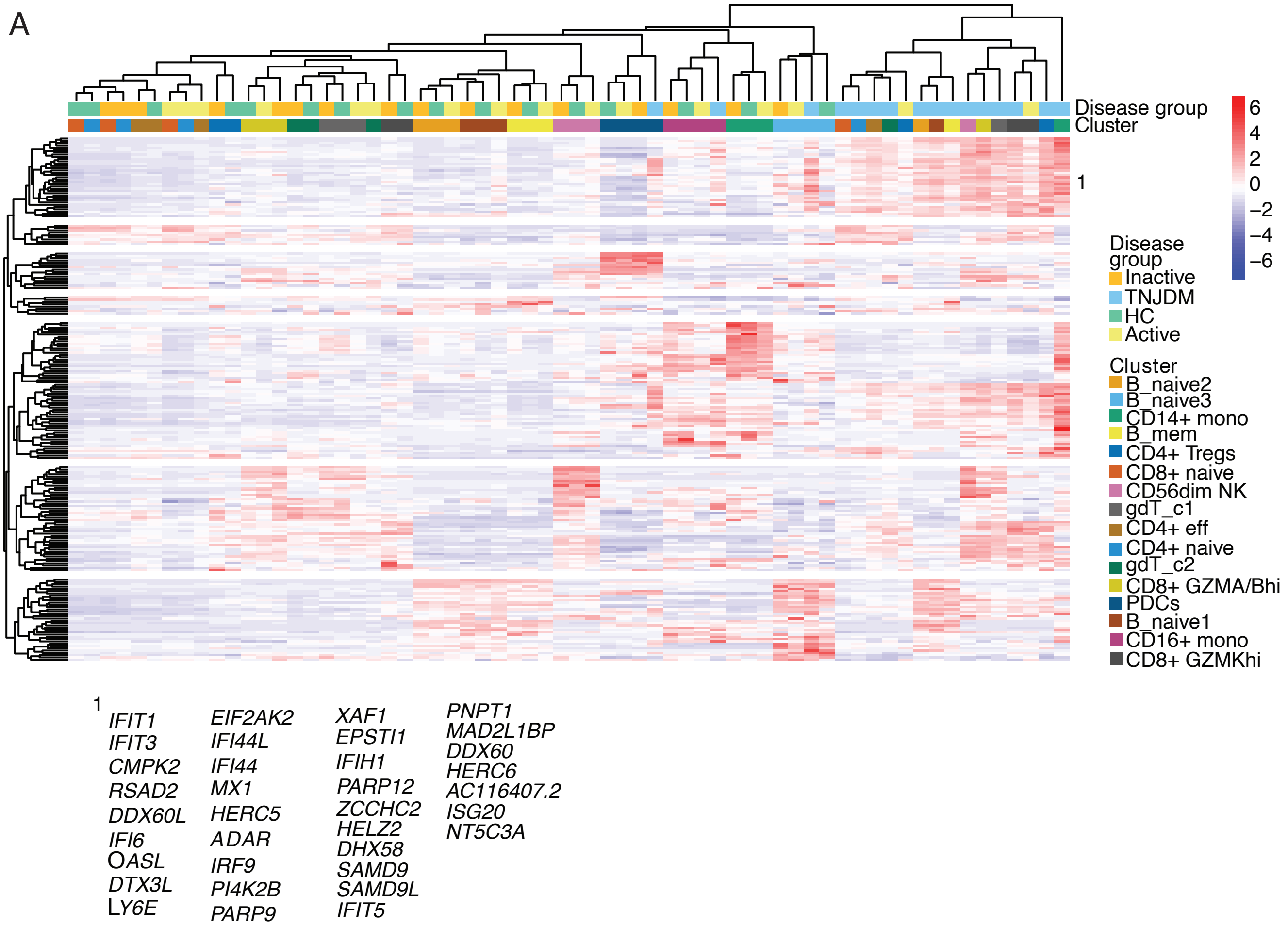

Supplemental Figure 5. (A) Heatmap of differentially expressed genes between TNJDM and HC from all cell types clustered by expression likeliness. The genes from cluster 1 were used to calculate the IFN score

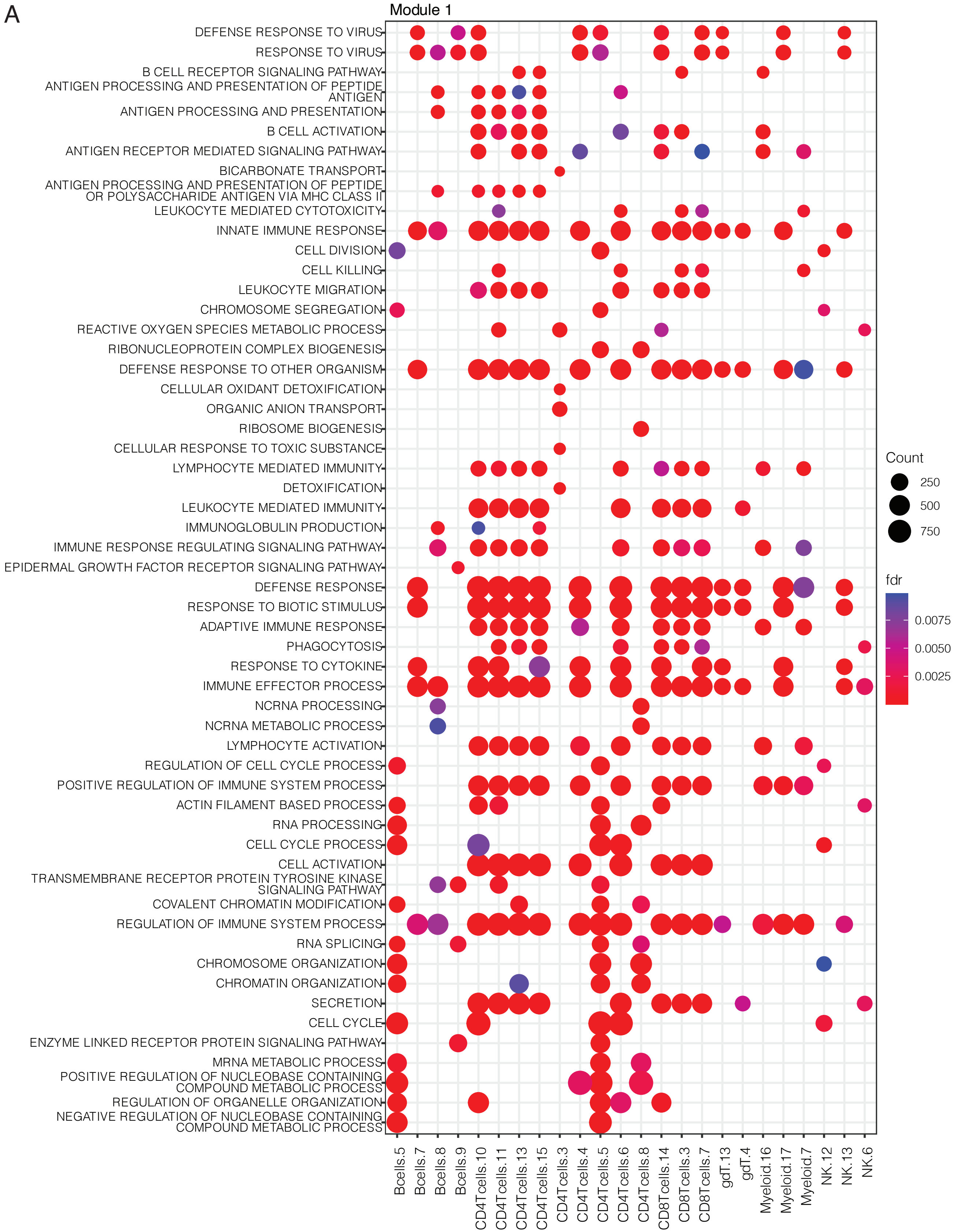

Supplemental Figure 6: (A) Gene set enrivhment results of GO terms for programs in Module 1 (FDR < 0.01).

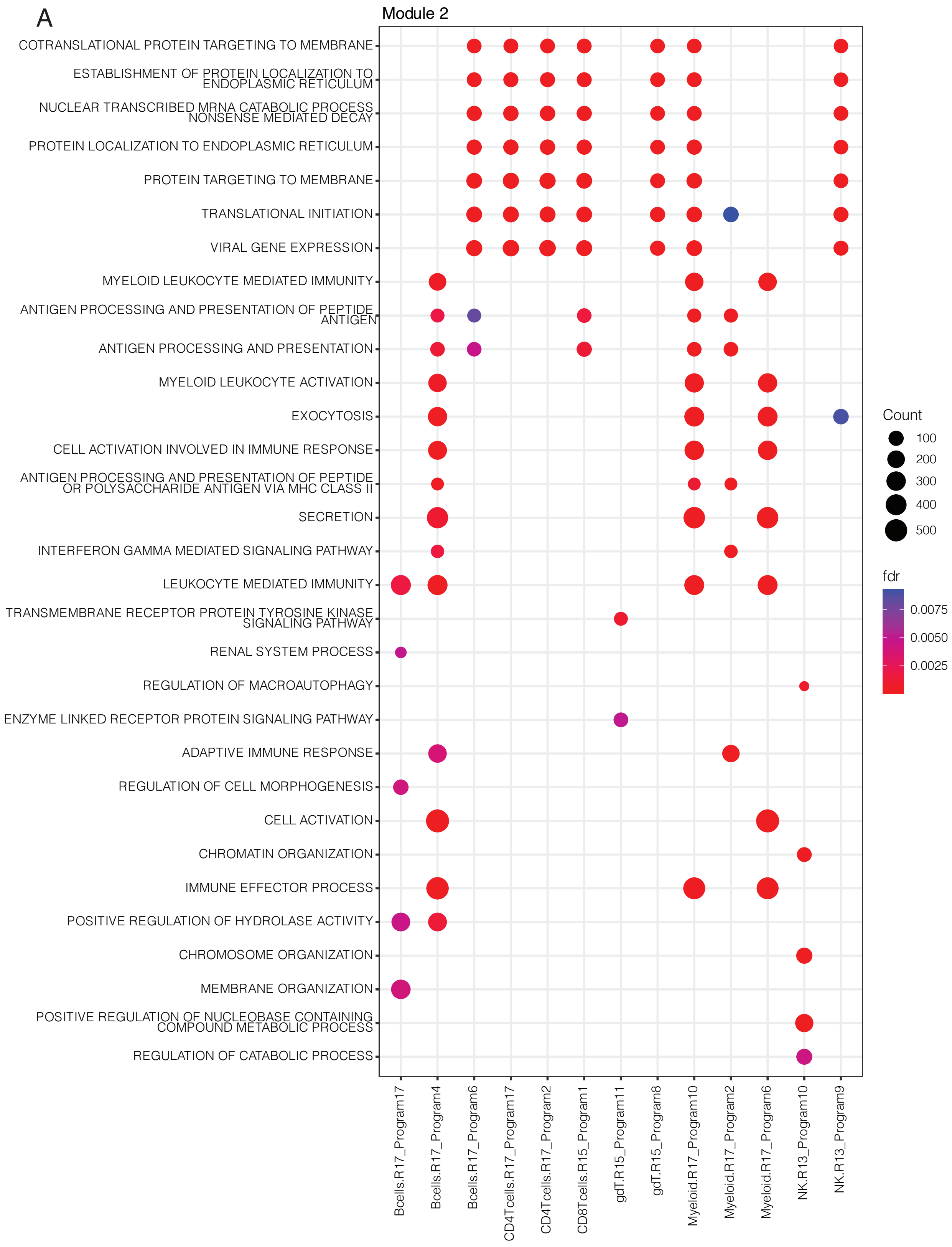

Supplemental Figure 7: (A) Gene set enrivhment results of GO terms for programs in Module 2 (FDR < 0.01).

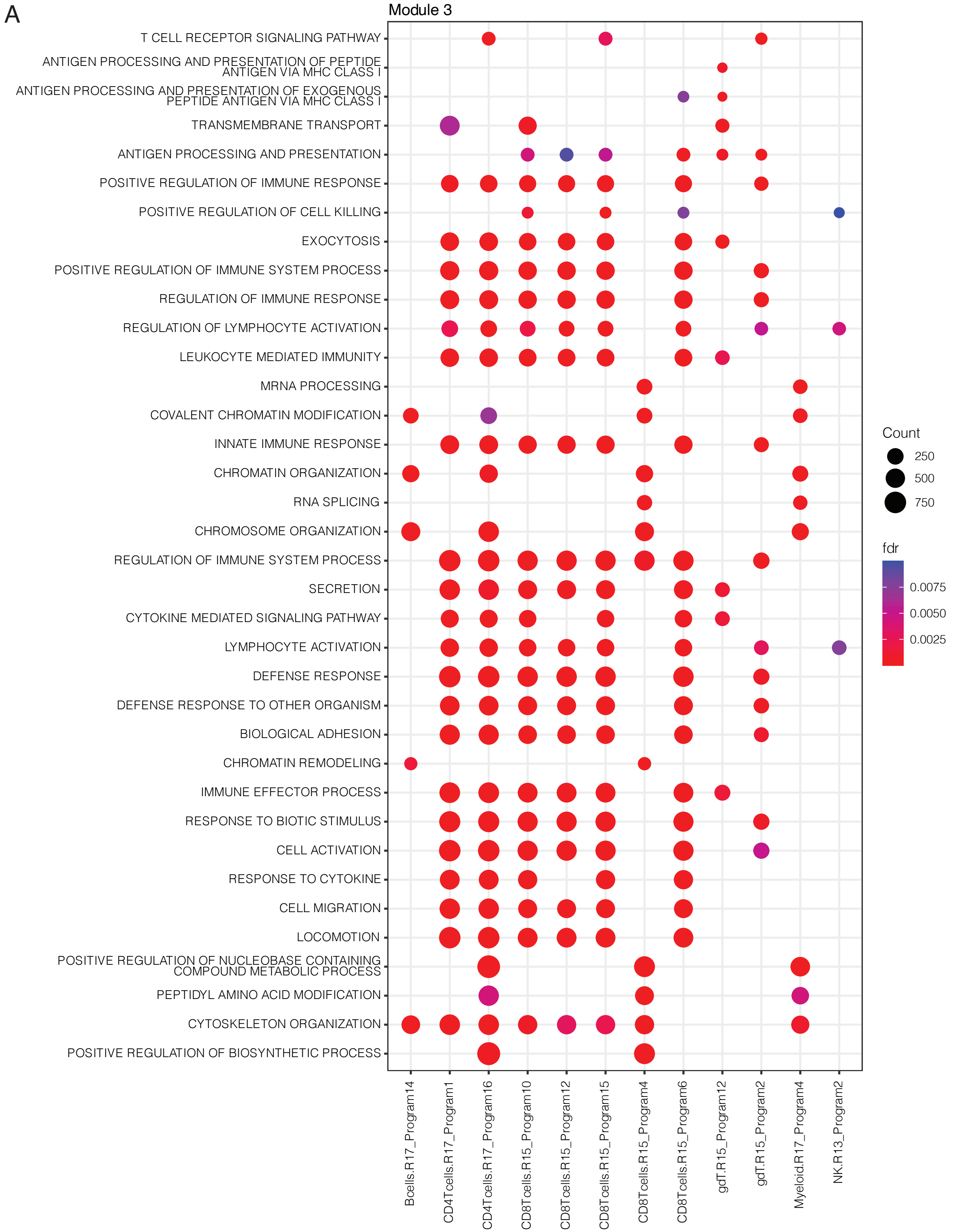

Supplemental Figure 8: (A) Gene set enrivhment results of GO terms for programs in Module 3 (FDR < 0.01).

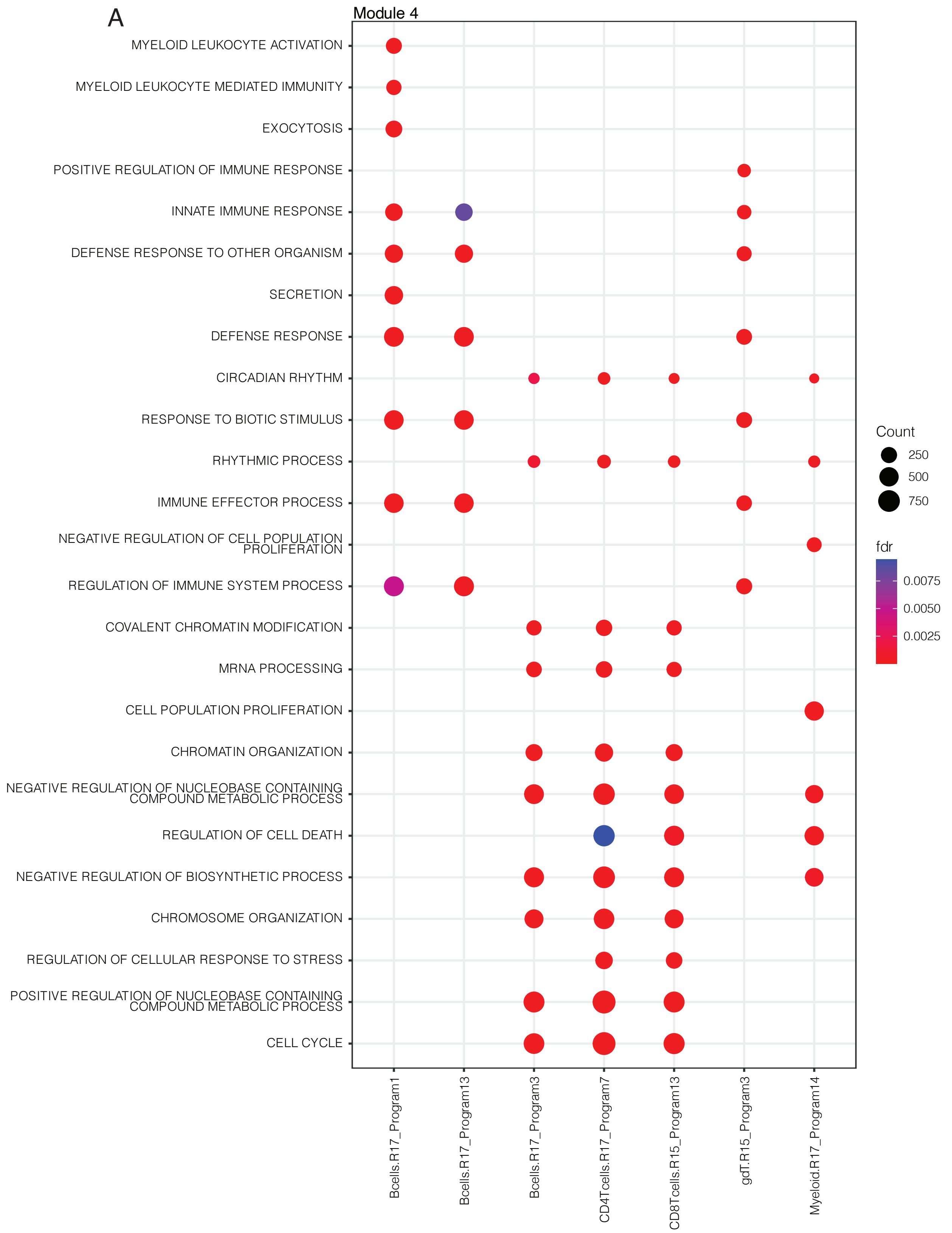

Supplemental Figure 9: (A) Gene set enrivhment results of GO terms for programs in Module 4 (FDR < 0.01).

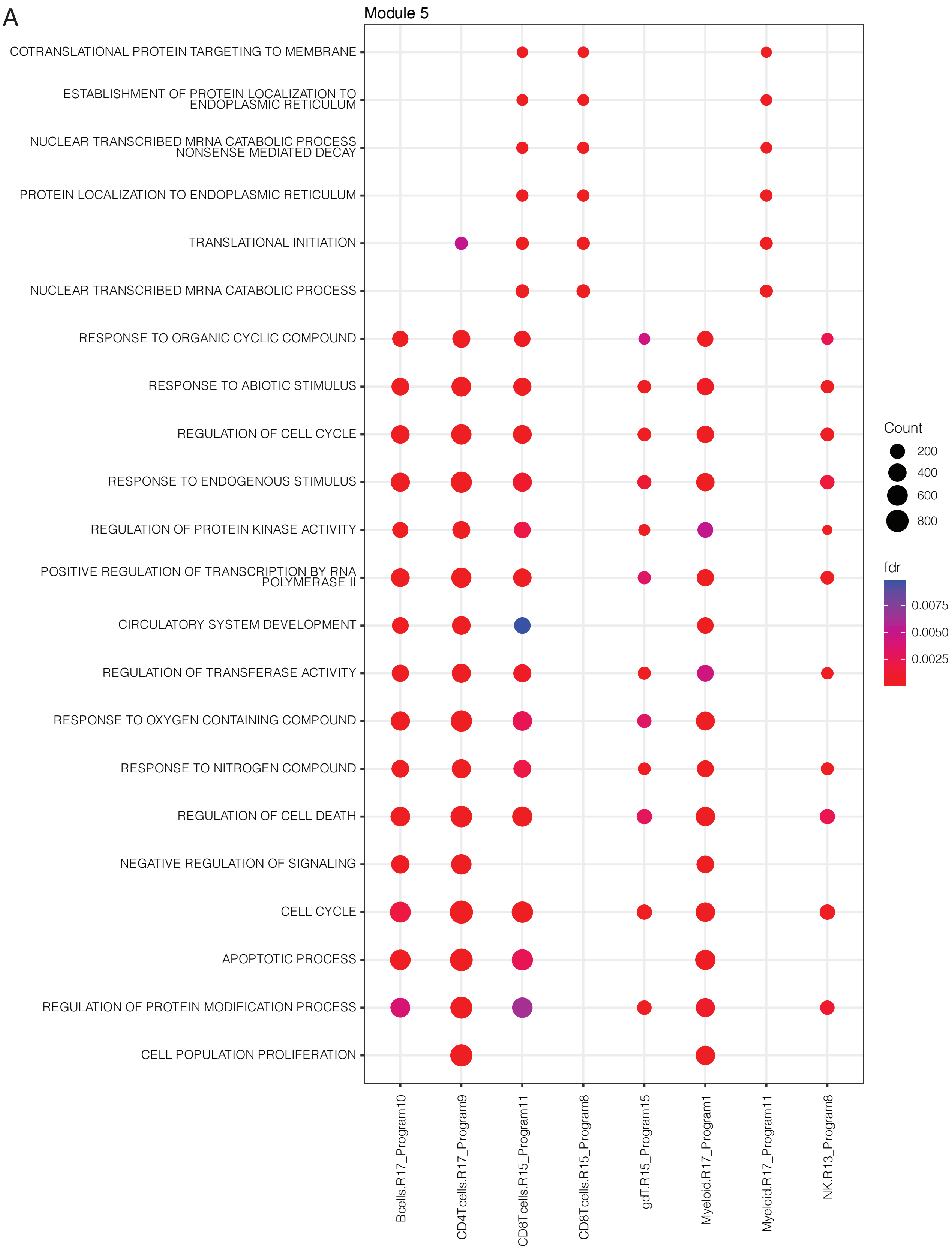

Supplemental Figure 10: (A) Gene set enrivhment results of GO terms for programs in Module 5 (FDR < 0.01).

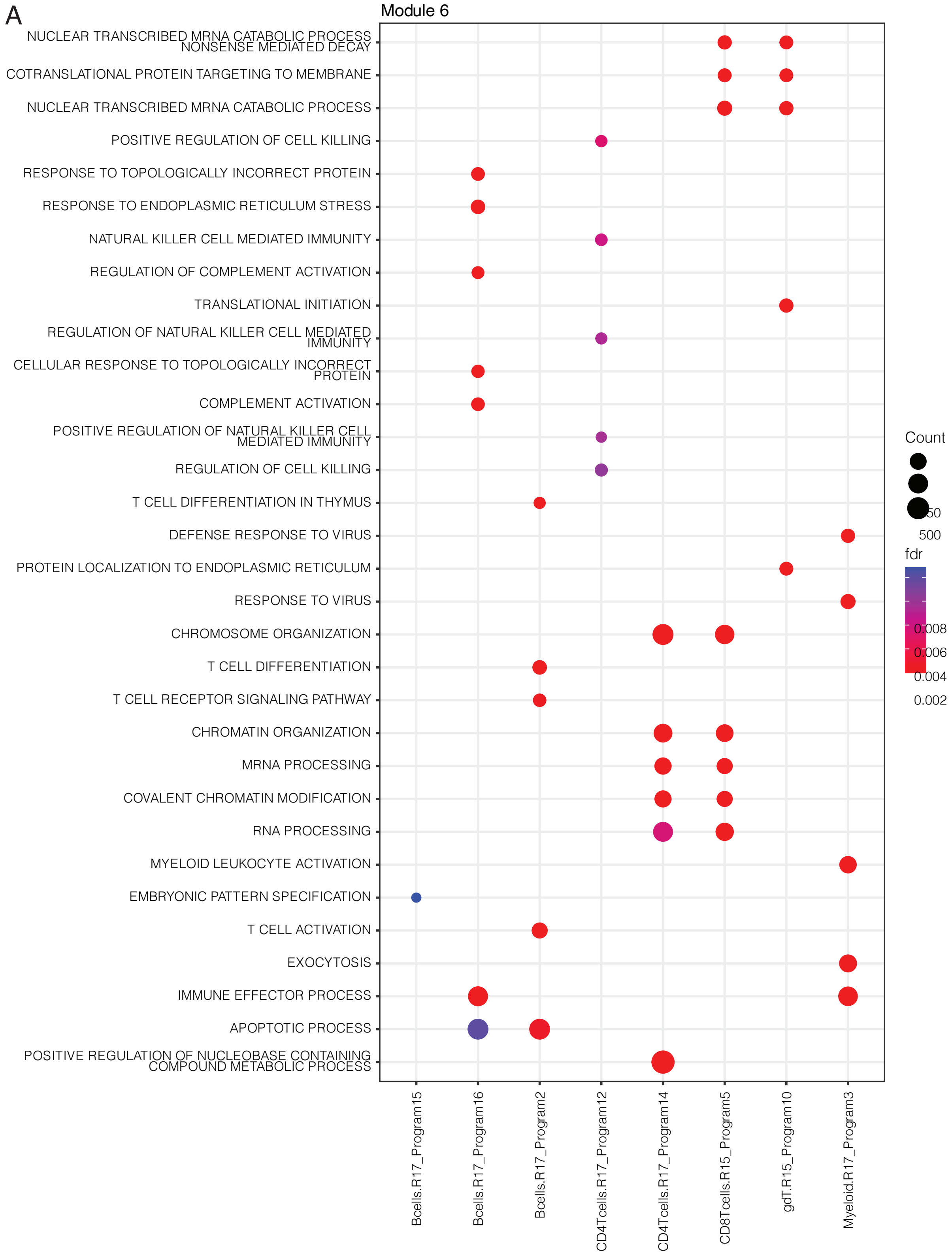

Supplemental Figure 11: (A) Gene set enrivhment results of GO terms for programs in Module 6 (FDR < 0.01).

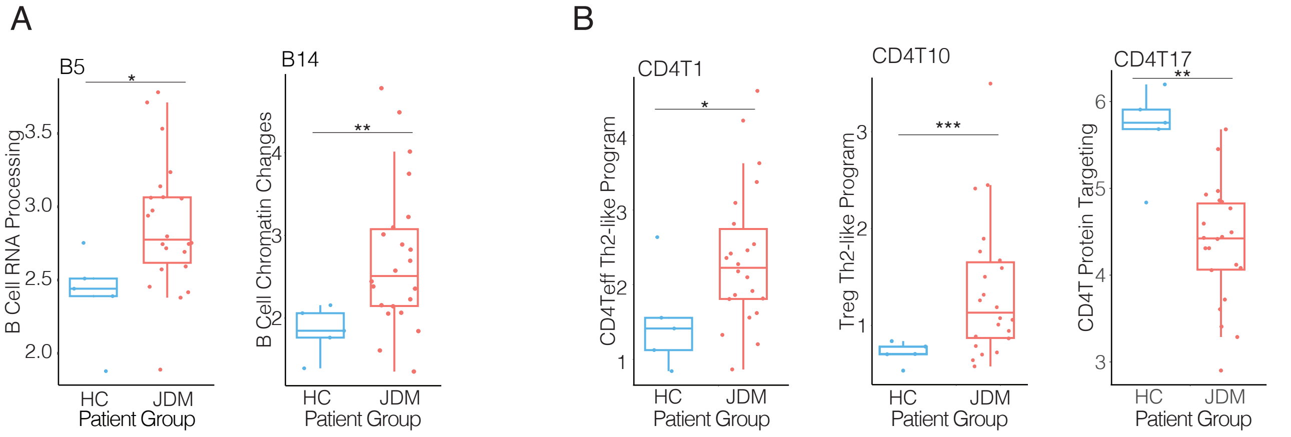

Supplemental Figure 12 (A-B) Mean patient expression of JDM-associated programs (t-test, p<0.05) in B **(A)** and CD4T **(B)** cell compartments, respectively (*p<0.05, **p<0.01, ***p<0.001).

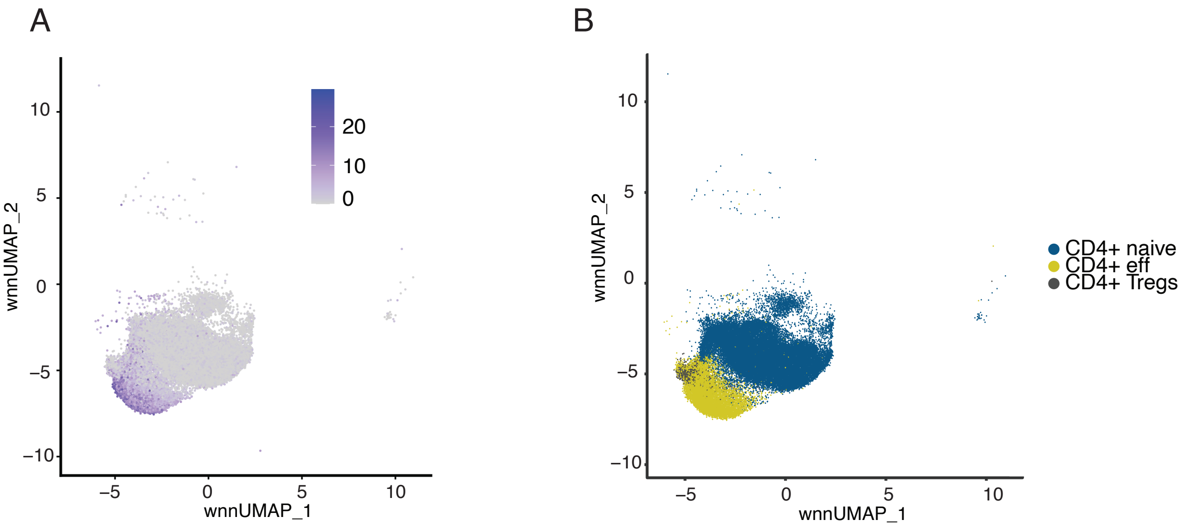

Supplemental Figure 13 (A-B) UMAPs of CD4T cells showing expression of NMF program CD4T1 **(A)** in subcluster corresponding to CD4+ effector cells **(B)**.

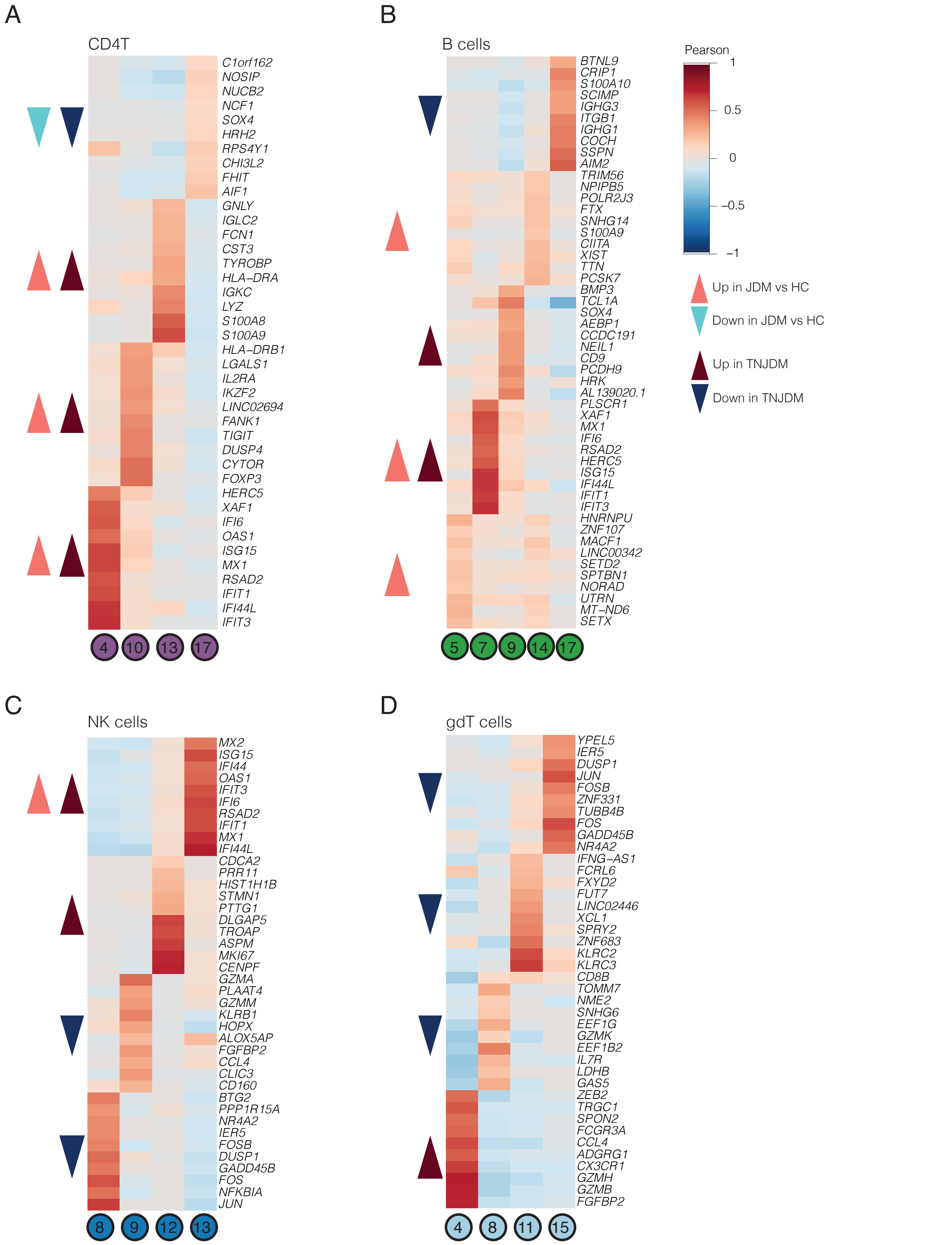

Supplemental Figure 14 (A-D) Heatmaps showing top 10 marker genes for selected disease-associated programs for the indicated cell type. Colored according to Pearson correlation between gene expression and program expression in the indicated cell type. Arrows indicate whether a given program is expressed higher or lower in a specific subset of patients.

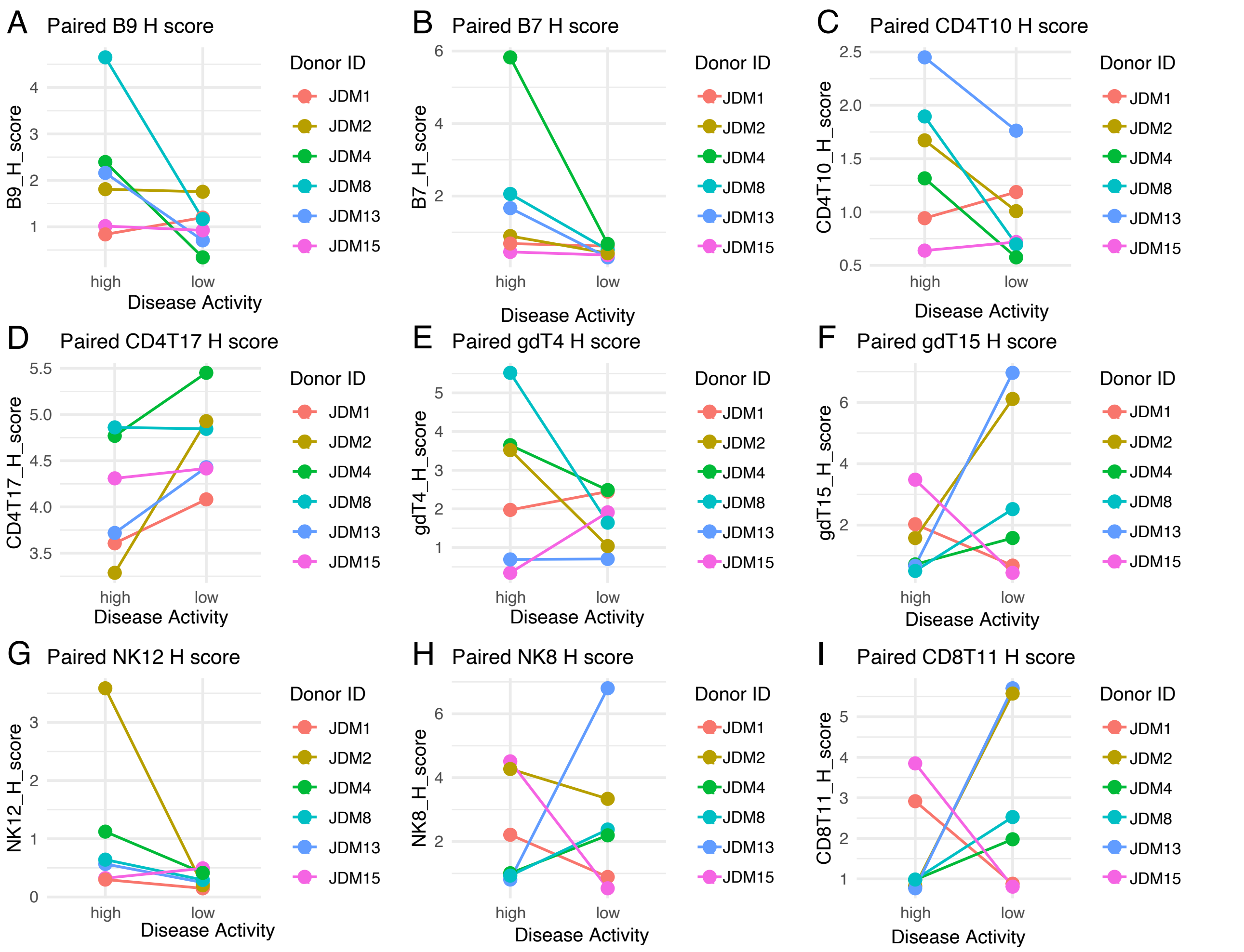

Supplemental Figure 15 (A-I) GEP scores for longitudinal samples collected at an individual's high and low disease activity point. Individuals are labelled using the same Donor ID used throughout the paper. Changes in expression within individuals trended, but in this subset of patients with longitudinal samples, there was insufficient statistical power to quantify statistical significance given disease activity heterogeneity across these 6 individuals.

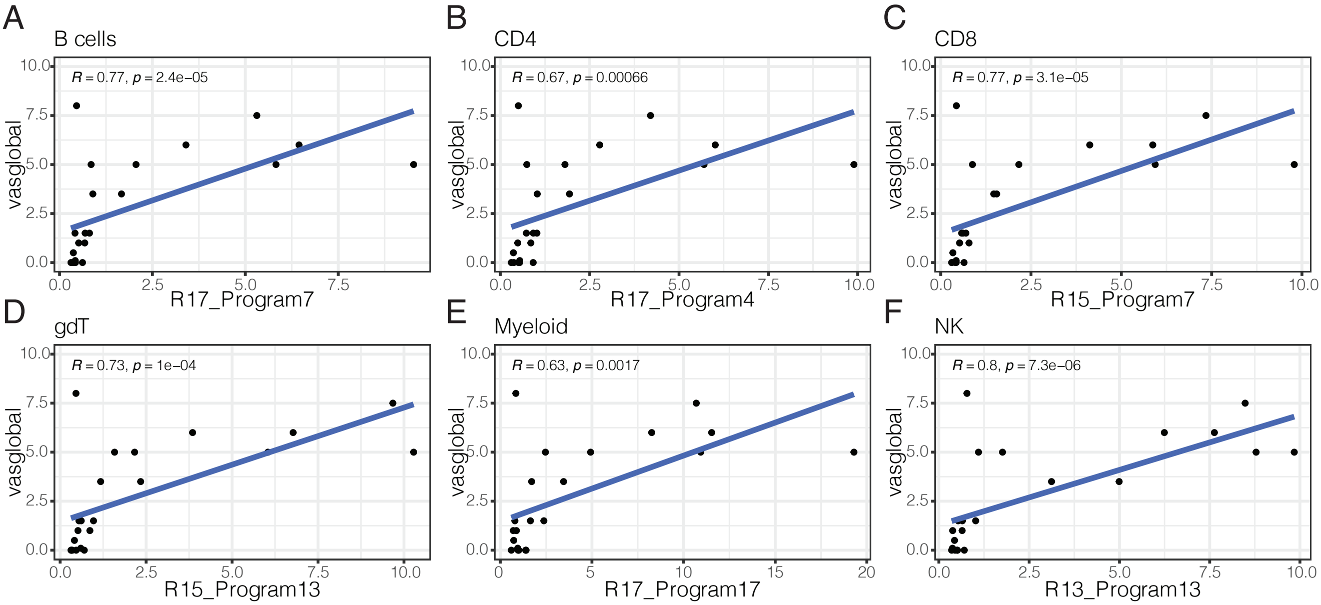

Supplementary Figure 16: (A-F) Scatter plots showing mean sample expression (n=27) of type I interferon response programs in each corresponding cell type (Pearson).

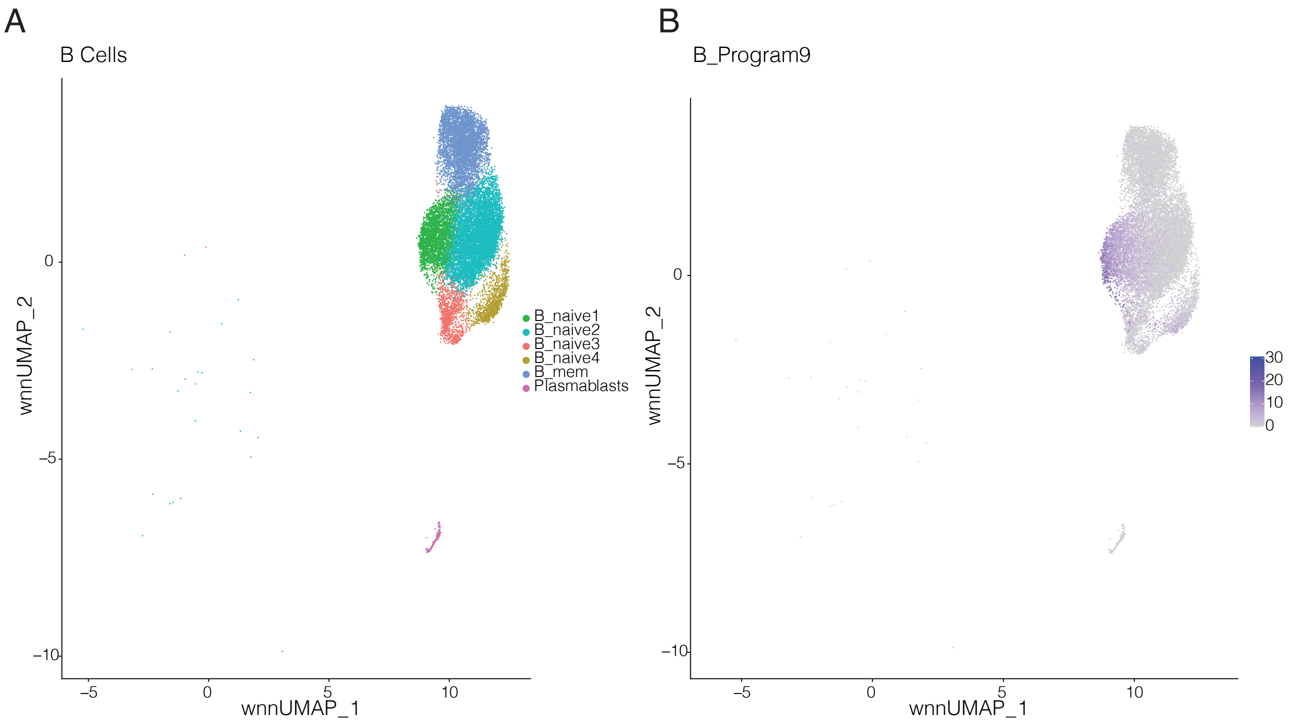

Supplemental Figure 17 (A) wnnUMAP of B cell subsets **(B)** wnnUMAP showing expression of program B9 in B cells.

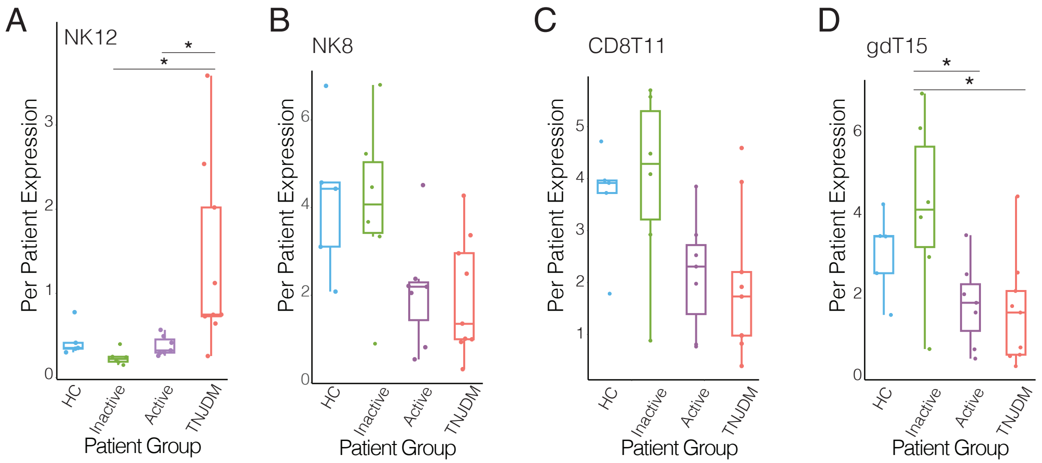

Supplemental Figure 18 (A-D) Mean patient expression of disease activity associated programs (4-way ANOVA, p<0.05) in Module 5 (*p<0.05 Post-hoc pairwise Tukey test).

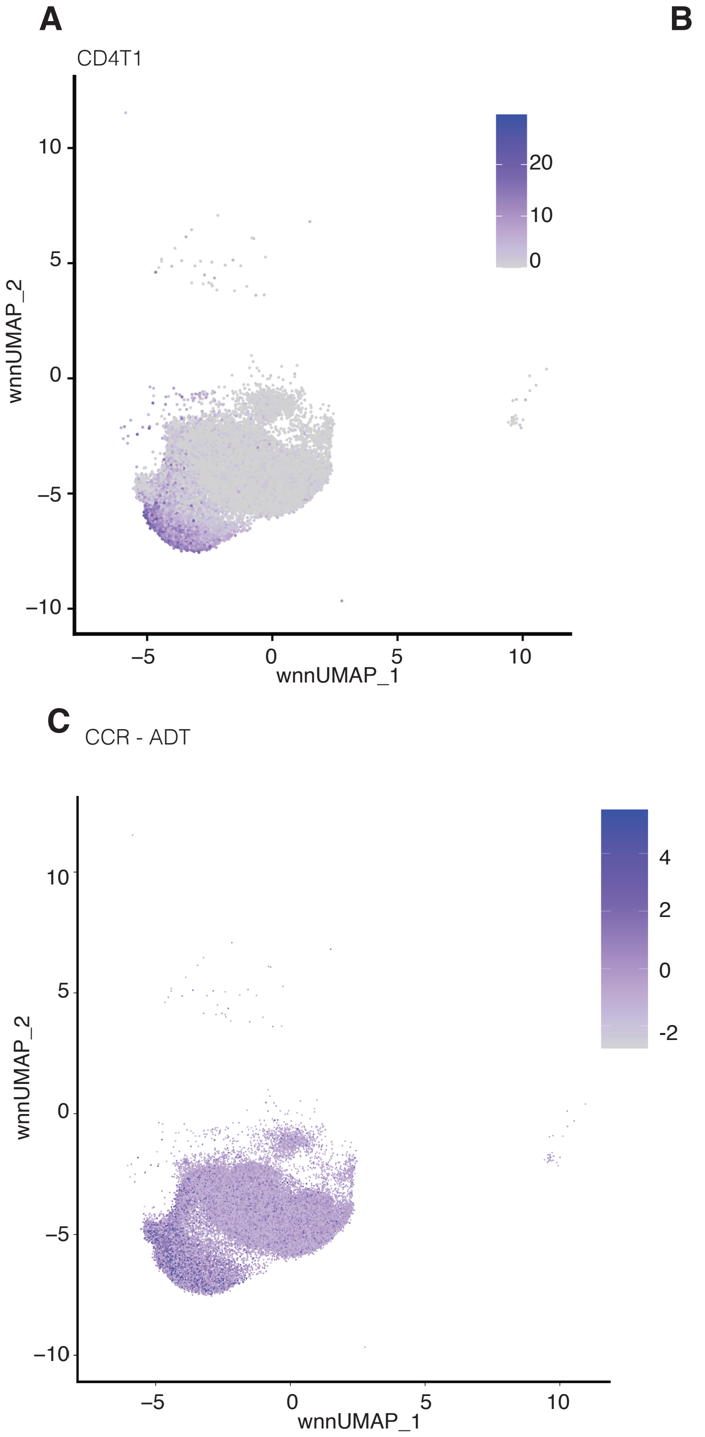

Supplemental Figure 19 (A-C) wnnUMAPs showing normalized expression of GEPs CD4T1 and CD4T10 **(A-B)** with co-expression of surface protein CCR4 **(C)**.

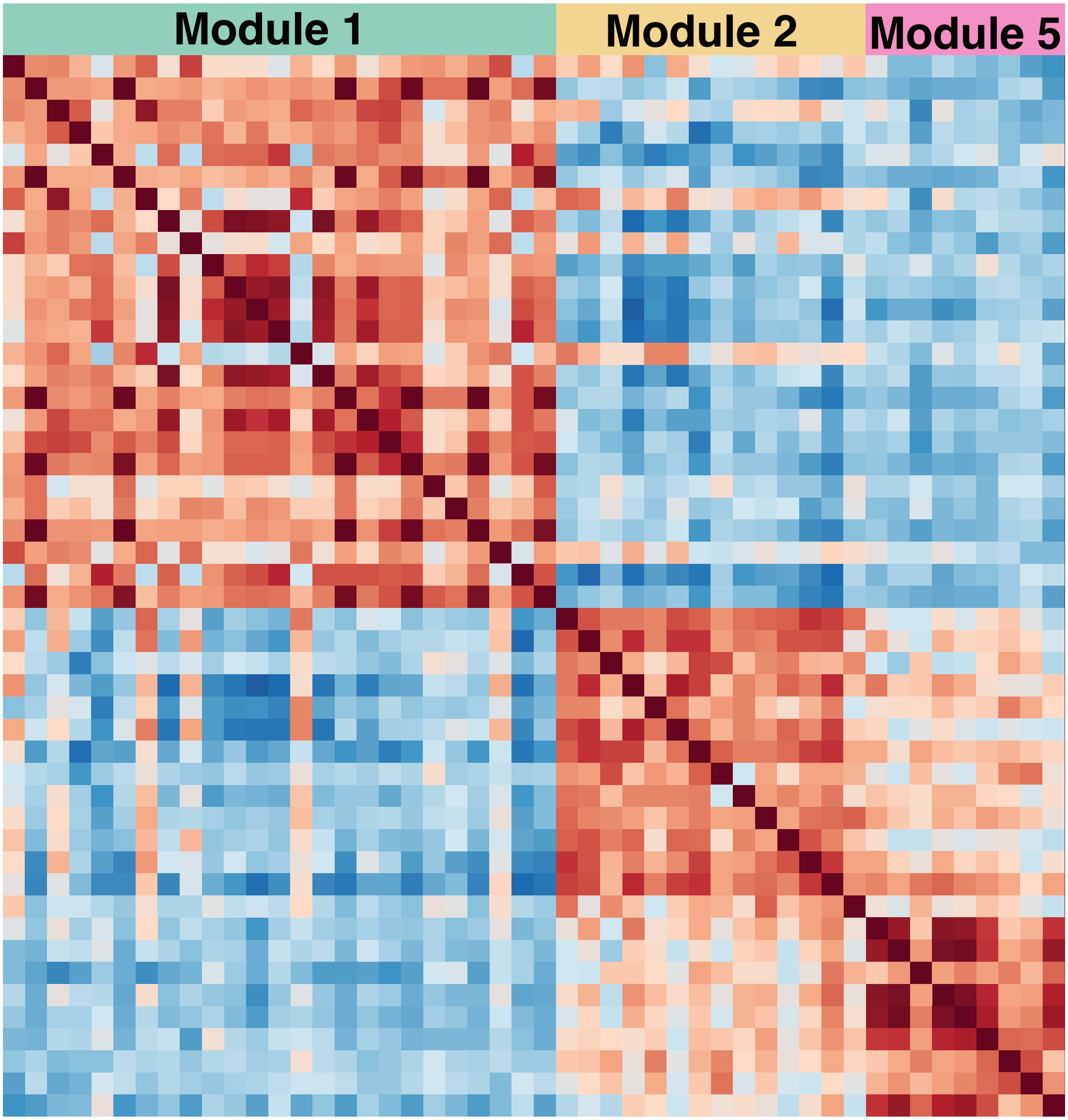

Supplemental Figure 20. (A) Subset of Modules 1, 2, and 5 from original heatmap in Figure 4B highlighting the negative correlations.

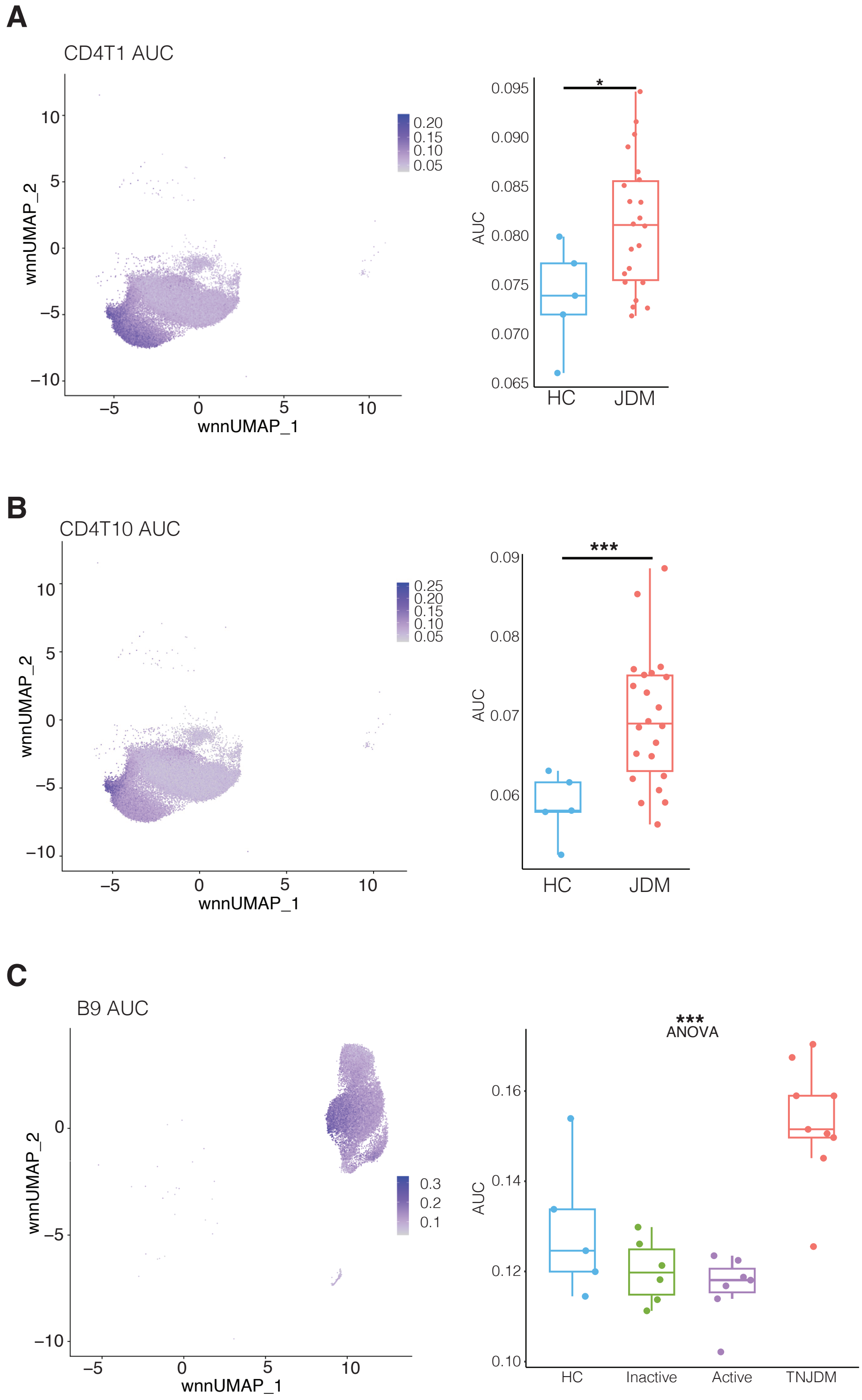

Supplemental Figure 21 (A-B) Single-cell expression of proxy GEP metric calculated using AUCell in the originaldataset and quantification of proxy program expression for each patient comparing HC (n=5) to JDM (n=22) (t-test: *p<0.05, ***p<0.001). **(C)** Single-cell expression of proxy GEP metric calculated using AUCell in the original dataset and quantification of proxy program expression for each patient comparing HC (n=5), Inactive JDM (n=6), Active JDM (n=7), and TN JDM (n=9) (4-group ANOVA ***p<0.001).

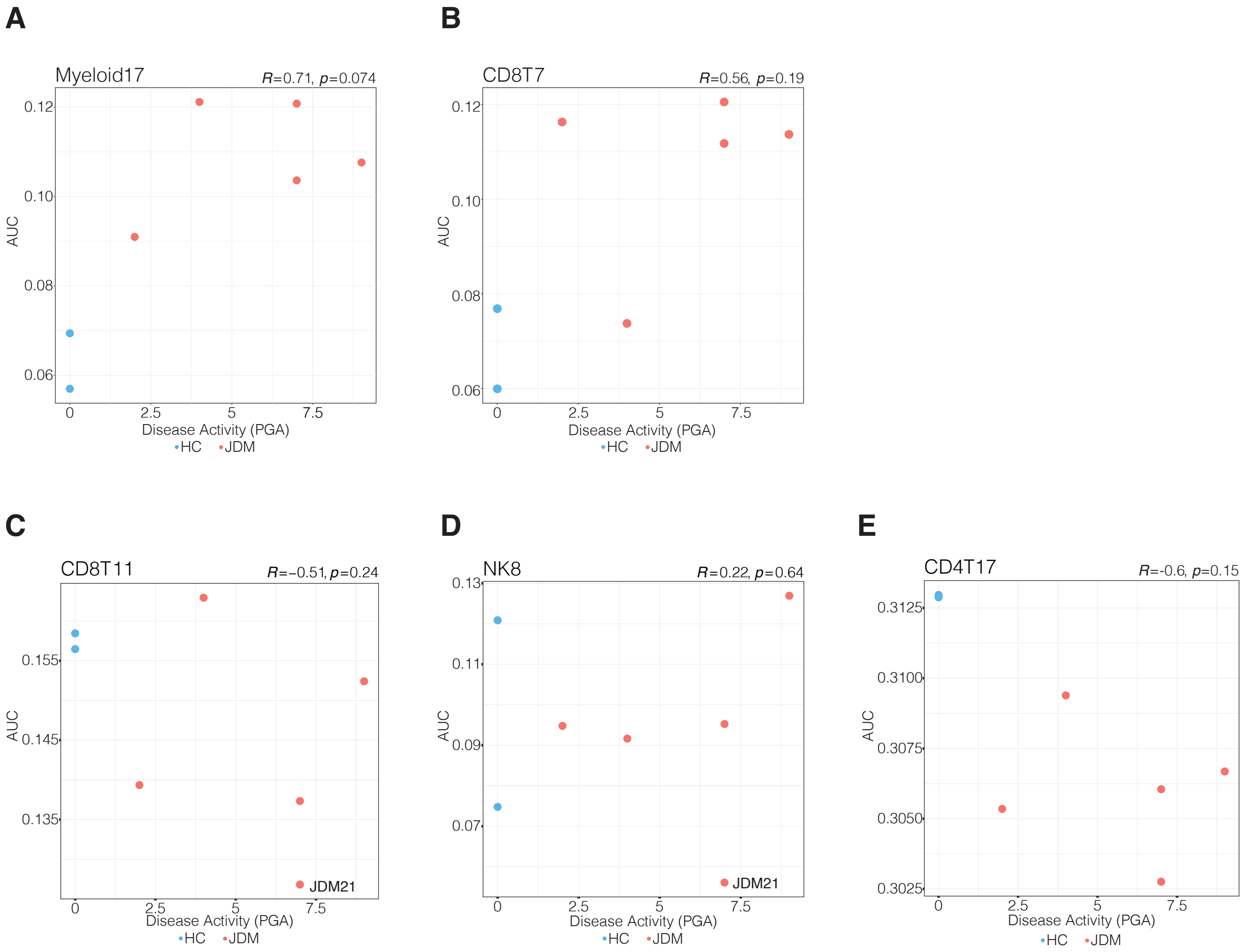

Supplemental Figure 22 (A-B) Scatterplots correlating disease activity (PGA) with AUCell scores for proxy IFN programs, Myeloid17 and CD8T7, in independent dataset (Spearman). **(C-E)** Scatterplots correlating disease activity (PGA) with AUCell scores for proxy CD8T11, NK8, and CD4T17 programs in independent dataset (Spearman). The cell death regulatory programs previously found to be lowest in active and treatment-naive JDM, CD8T11 and NK8, were not significantly correlated in the independent dataset, though the single treatment-naïve patient (JDM21) exhibited the lowest scores for both programs. CD4T17 exhibited a negative trend with disease activity that did not meet the threshold for significance

A

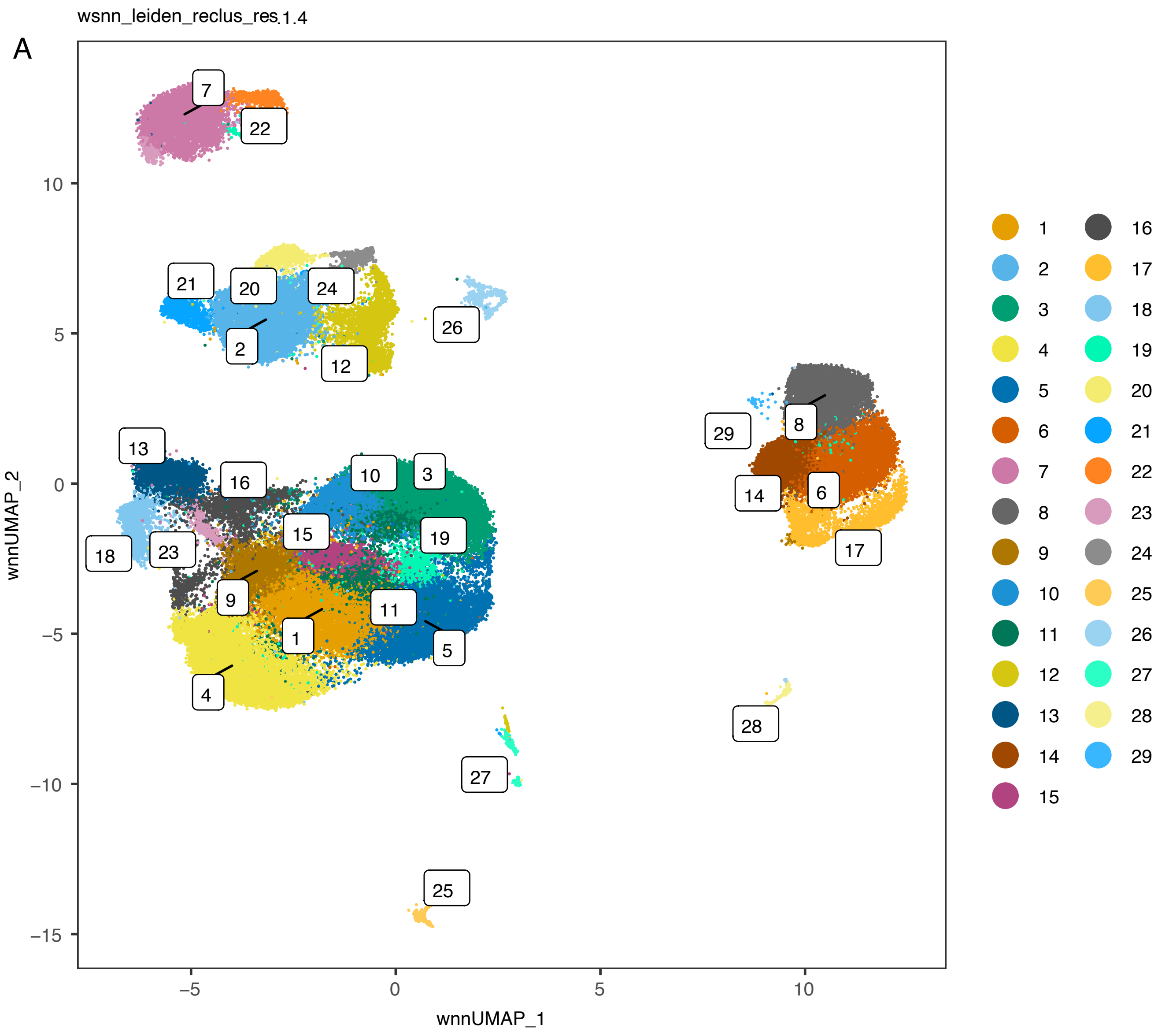

Supplemental Figure 23. (A) Original wnnUMAP, using Leiden clustering with a resolution of 1.4

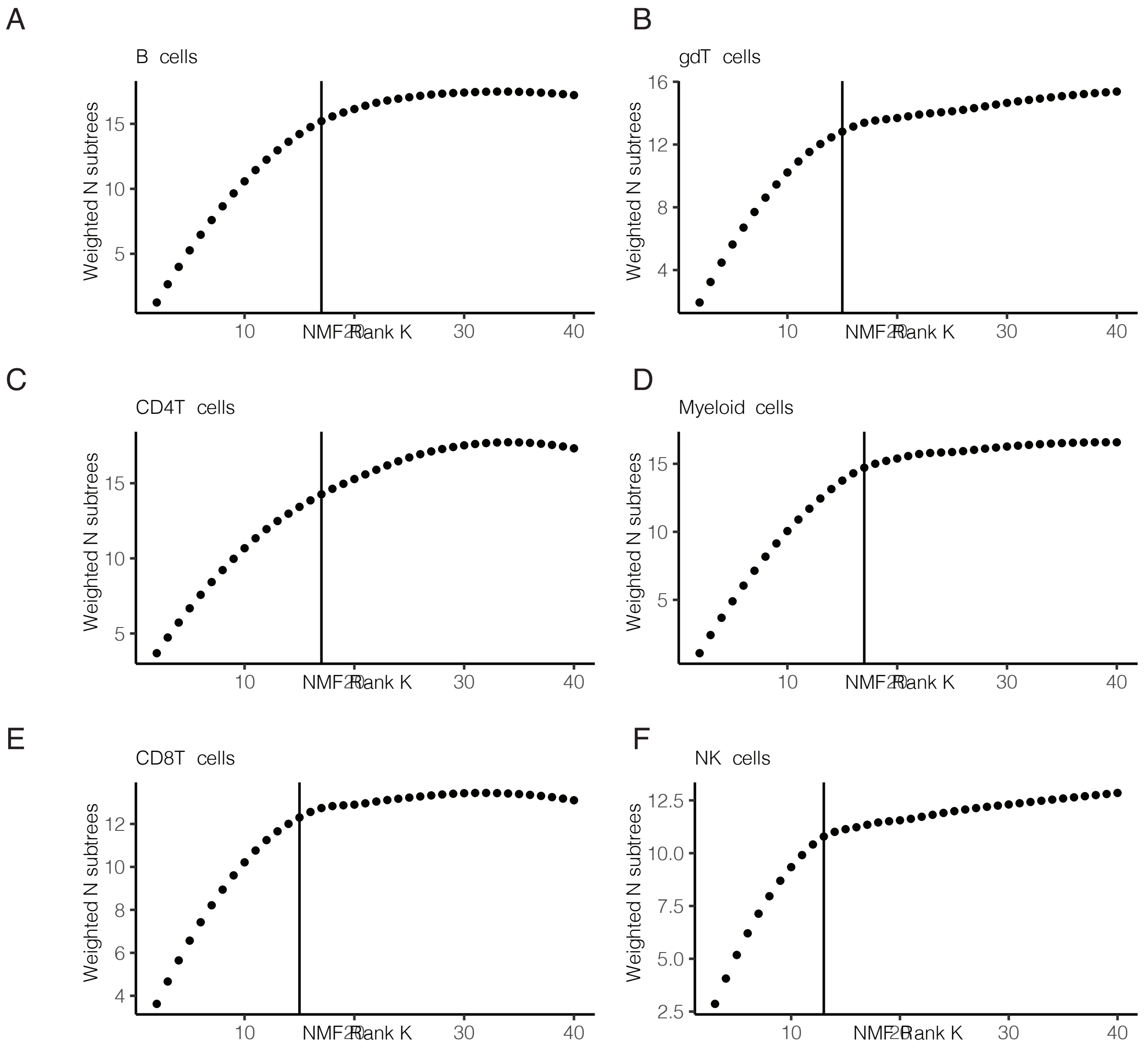

Supplemental Figure 24. (A-F) Elbow plots for rank selection for NMF ran on each major cell type. K was chosen as inflection point on scatter plot where rank maximizes weighted subtree metric.
