# Supplementary material for: Coordinated immune dysregulation in juvenile dermatomyositis revealed by single-cell genomics
Source: JCI Insight. 2024 May 14;9(12):e176963. doi: 10.1172/jci.insight.176963 (PMC11383589; doi:10.1172/jci.insight.176963)
Supplement: Supplemental table 1 [file jciinsight-9-176963-s179.pdf]

Supplemental Table 1. Clinical cohort disease characteristics.

	TNJDM (N=9)	Active (N=7)	Inactive (N=6)	HC (N=5)
**Sex**				
Female	3 (33.3%)	4 (57.1%)	5 (83.3%)	2 (40.0%)
Male	6 (66.7%)	3 (42.9%)	1 (16.7%)	3 (60.0%)
**Age (years)**				
Median [min-max]	7.0 [2.0-15]	13 [5.0-18]	16 [5.0-18]	3.0 [1.0-16]
**Prednisolone treatment — no. (%)**	0 (0%)	1 (14.3%)	0 (0%)	
**Methyl prednisolone treatment — no. (%)**	0 (0%)	1 (14.3%)	0 (0%)	
**Methotrexate treatment — no. (%)**	0 (0%)	4 (57.1%)	0 (0%)	
**HQL treatment — no. (%)**	0 (0%)	4 (57.1%)	0 (0%)	
**IVIG treatment — no. (%)**	0 (0%)	1 (14.3%)	0 (0%)	
**Physician Global VAS**				
Median [min-max]	5.0 [1.5-7.5]	1.5 [0.50-8.0]	0 [0-0.10]	
**Muscle VAS**				
Median [min-max]	4.0 [0.50-8.5]	0.50 [0-3.5]	0 [0-0]	
**Cutaneous VAS**				
Median [min-max]	4.0 [1.5-7.0]	1.0 [0-7.0]	0 [0-0.20]	
**Patient/Parent Global VAS**				
Median [min-max]	5.0 [2.0-8.2]	2.0 [0-8.0]	0 [0-0.40]	
Missing	2 (22.2%)	0 (0%)	0 (0%)	
**CDASIACT**				
Median [min-max]	16 [4.0-35]	2.0 [0-8.0]	0 [0-1.0]	
**CHAQ-score**				
Median [min-max]	1.0 [0-2.4]	0 [0-1.3]	0 [0-0.38]	
Missing	2 (22.2%)	0 (0%)	0 (0%)	
**MMT8-score**				
Median [min-max]	70 [59-76]	80 [67-80]	80 [79-80]	
Missing	4 (44.4%)	0 (0%)	0 (0%)	
**MSA**				
MDA5	1 (11.1%)	0 (0%)	0 (0%)	
NEG	1 (11.1%)	1 (14.3%)	1 (16.7%)	
NXP2	1 (11.1%)	4 (57.1%)	2 (33.3%)	
TIF1y	6 (66.7%)	2 (28.6%)	2 (33.3%)	
UNK	0 (0%)	0 (0%)	1 (16.7%)	
**Muscle enzyme elevation Present — no. (%)**	8 (88.9%)	3 (42.9%)	1 (16.7%)	

Overview of clinical characteristics from the 15 patients with JDM and 5 healthy controls, totalling 27 samples. Some patients had longitudinal sampling at different disease stages. Myositis specific antibodies were sent to Oklahoma Myositis Research Foundation for testing. TNJDM = treatment-naive JDM, Active = Active JDM defined by Physician global visual analog score (VAS) ≥ 0.5 and on medication, Inactive = Inactive JDM defined by physician global VAS < 0.5 and off medication, HC = healthy control, HQL = Hydroxychloroquine, VAS = Visual Analogue Scale, CDASIACT = Cutaneous Dermatomyositis Disease Area and Severity Index Activity Score, CHAQ = Childhood Health Assessment Questionnaire, MMT = Manual Muscle Testing, MSA = Myositis Specific Antibodies.
